# Behavioral ontogeny in a pelagic tunicate reveals developmental maturation and context-dependent modulation

**DOI:** 10.1016/j.isci.2026.118104

**Published:** 2026-11-04

**Authors:** Oleg Tolstenkov, Rodolfo da Silva Mazzarini Baldinotti, Sissel Norland, Anne Elin Aasjord, Daniel Chourrout, Marios Chatzigeorgiou

**Affiliations:** 1 Michael Sars Centre, Faculty of Science and Technology, University of Bergen, Bergen, Norway; 2 Department of Life Sciences, Imperial College London, London SW7 2AZ, UK

**Keywords:** tunicate, *Oikopleura dioica*, behavioral developmental plasticity, animal behavior, swimming

## Abstract

Behavioral ontogeny refers to the development of an individual’s behavior over its lifespan, shaped by a complex interplay between genes and the environment. While well studied in vertebrates, its presence in other chordates, including tunicates, the closest vertebrate relative, remains unclear. We constructed a behavioral atlas of the planktonic tunicate *Oikopleura dioica* across its life cycle using high-throughput tracking and unsupervised analysis. Swimming kinematics, exploration, and thigmotaxis increased with development. Adults exhibit a unique innovation, the house, a complex extracellular filtration structure encasing the trunk, which drastically alters locomotion and exploration compared to free-swimming individuals. Postural variation during ontogeny is captured by seven basic shapes *eigenoikopleuras*, with complex shapes becoming more frequent over time. Spatiotemporal embedding and hidden Markov modeling revealed that behavioral refinement arises from changes in motor modules and their transitions. House occupancy similarly shifts these modules. This continuous behavioral maturation parallels *Oikopleura*’s neotenic morphology, providing a quantitative framework for exploring ontogenic behavioral maturation in chordates.

## Highlights

• Behavioral complexity increases progressively across *Oikopleura* ontogeny
• Development reweights conserved motor modules rather than creating new ones
• Seven *eigenoikopleuras* explain most postural variation across life stages
• House occupancy reshapes locomotion and behavioral state organization

## Graphical abstract

**Figure:**
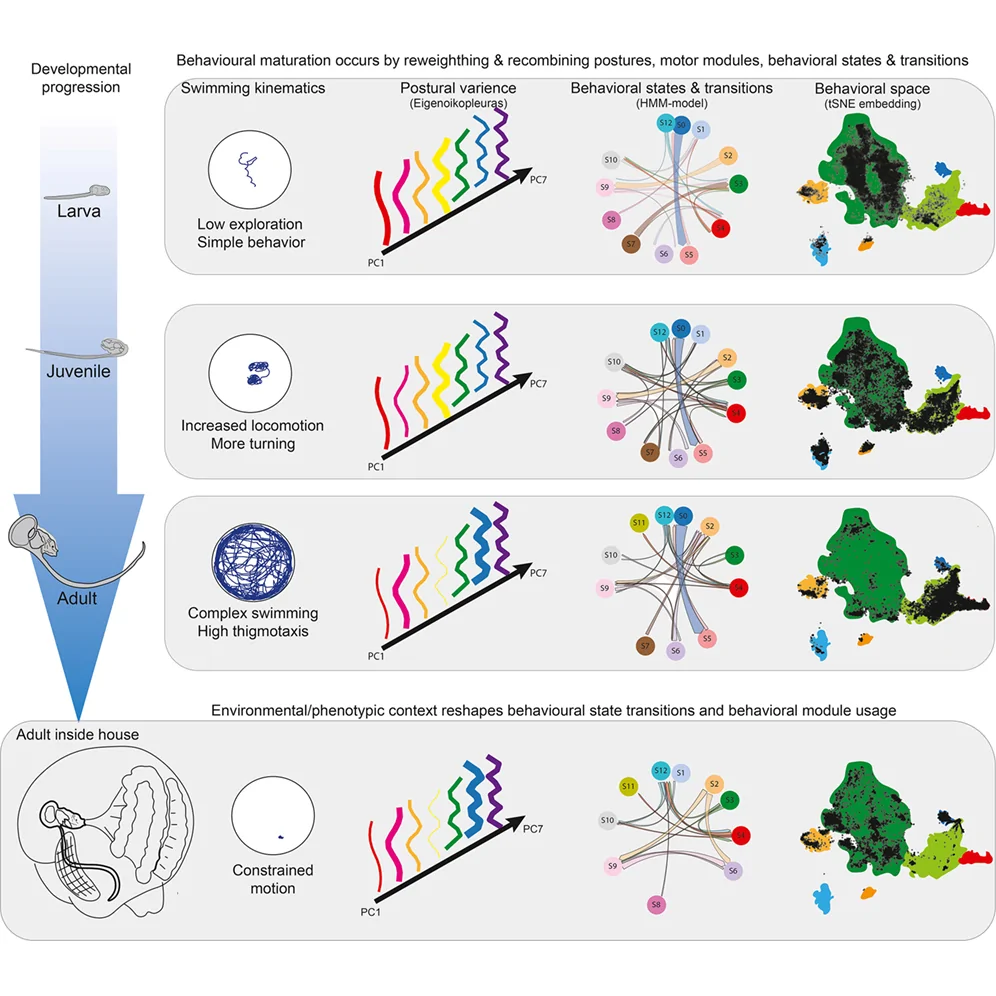

## Teaser

Zoology; Evolutionary biology; Developmental biology

## Introduction

Behavioral development is a complex process, influenced by an animal’s genetic traits, early experiences, ongoing interactions and various environmental factors.^1^^,^^2^ We are most familiar with developmental change of the behavioral repertoire of our own species (*Homo sapiens*), where certain pivotal changes with far-reaching impact, termed “behavioral cusps” (e.g., the ability to crawl, walk, and speak) dramatically alter our interaction with our environment and other humans.^3^^,^^4^

Behavioral changes across development can arise from both “ontogenetic maturation” and “behavioral developmental plasticity.” Ontogenetic maturation refers to age-dependent refinement of behavior driven by the progressive maturation of morphology, biomechanics, and sensorimotor circuits.^5^^,^^6^ By contrast, behavioral developmental plasticity describes cue-dependent modifications of developmental trajectories, whereby experience or environmental conditions shape behavior across development.^5^^,^^6^

Behavioral change across ontogeny has been studied in a limited number of organisms including the roundworm *Caenorhabditis elegans*,^7^ the freshwater copepod *Sinodiaptomus sarsi*,^8^ the fish *Danio rerio*,^9^^,^^10^ and *Poecilia formosa* (amazon mollies).^11^ These animals show surprising behavioral plasticity despite the conservative changes that take place in the nervous system during their post-embryonic ontogeny.^12^^,^^13^^,^^14^^,^^15^

These studies cover a minute fraction of the animal diversity on our planet; therefore, there is a significant gap in our understanding of behavioral developmental plasticity across most branches of the phylogenetic tree of life. Vertebrates belong to the phylum Chordata, which is composed of three groups (cephalochordates, tunicates, and vertebrates).^16^^,^^17^ The discovery that cephalochordates are basal within chordates and thus the tunicates are the sister group of vertebrates, suggesting that the adult ancestral chordate animals were free-swimming organisms in contrast to the ascidians which have a free-swimming larva with a rich behavioral repertoire^18^ (also reviewed in Piekarz and Stolfi^19^) and a sessile filter-feeding (yet behaving) adult.^20^ This finding brought renewed interest in appendicularians, the only tunicates that retain a free-swimming, chordate body plan throughout their life^21^^,^^22^ (Figure 1A), likely representing the ancestral tunicate condition, considering their position as the sister group to all other tunicates.^16^^,^^17^^,^^25^^,^^26^

**Figure 1:**
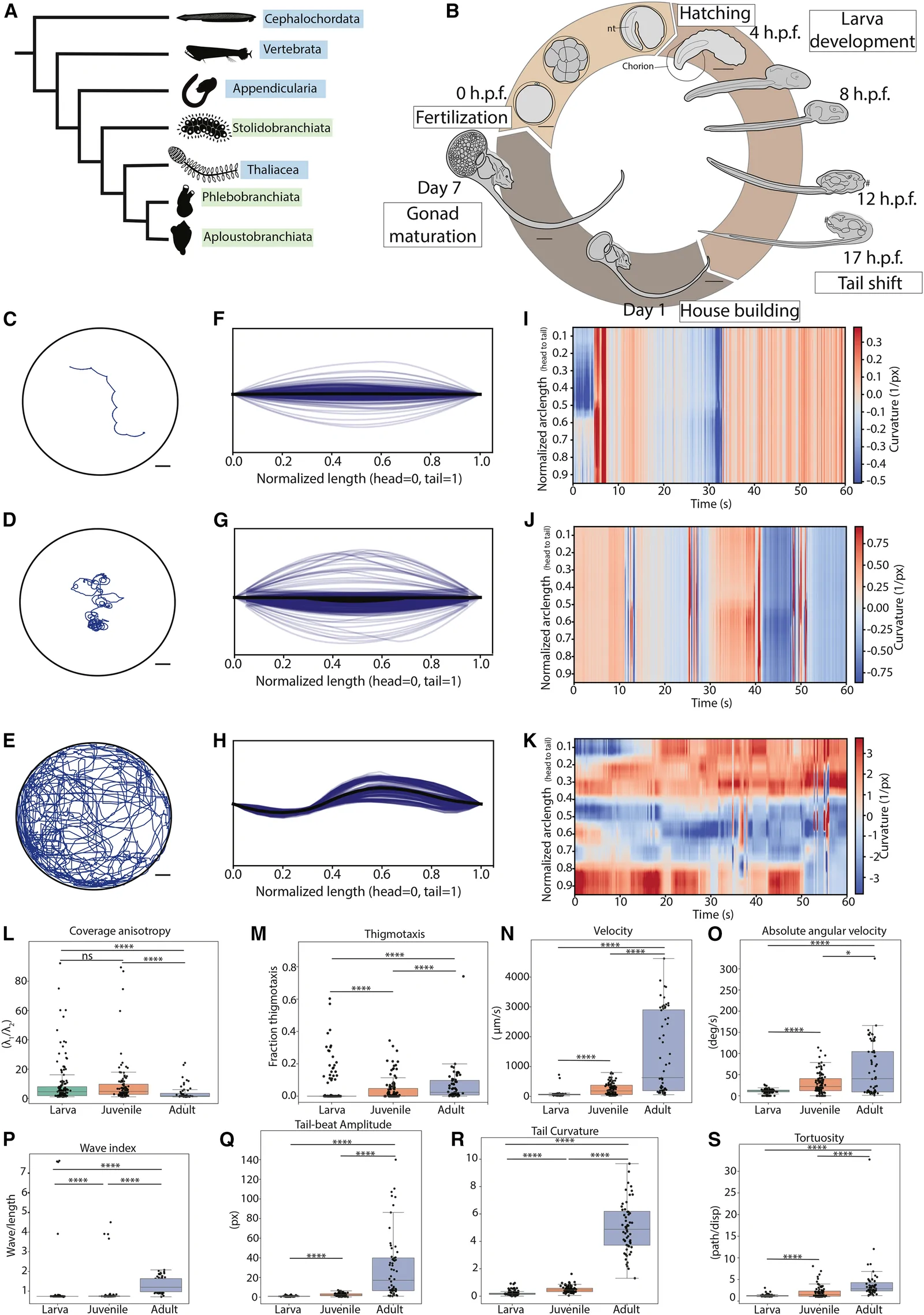
Developmental changes in *Oikopleura dioica* locomotor behavior (A) Phylogenetic position of appendicularia within chordates. Note that basal groups are the free-swimming cephalochordates within chordates and the free-swimming appendicularians within tunicates (modified from Giribet^23^). (B) Schematic representation of *Oikopleura dioica* life cycle (modified from Ferrandez-Roldan et al.^24^). Embryonic development begins with oocyte fertilization and concludes with larval hatching through rupture of the chorion (∼4 h.p.f. at 13°C). Larval development lasts for approximately 13 h (at 13°C) and culminates in tail-shift, a process during which the larval tail rotates by 120°. Note the change in the position of the mouth (hash sign) relative to the tail following this transition in juvenile animals. Over the subsequent ∼6 days (at 13°C), juveniles feed, grow, and differentiate into mature males or females, which spawn gametes and thereby complete the life cycle. Scale bars at 0 and 4 h.p.f. correspond to 50 μm, while scale bars at day 1 and day 5 correspond to 250 and 500 μm, respectively. (C–E) Representative 60 s trajectory traces. (C) Larval stage showing minimal spatial exploration. (D) Juvenile stage displaying increased spatial coverage. (E) Adult stage exhibiting extensive exploratory behavior with complex path structure. For all three images, the scale bars correspond to 1 mm. (F–H) Normalized centerline overlays (*n* = 200 traces per stage; gray = individual centerlines, dark blue = mean). Head positioned at (0.0) and tail at (1.0) after normalization. (F) Larva shows extended, relatively straight body. (G) Juvenile: moderate body curvature. (H) Adult: pronounced mid-body bend. Head positioned at (0,0) and tail at (1,0) after normalization. (I–K) spatiotemporal curvature heatmaps over 60 s. *y* axis: normalized body length (0 = head, 1 = tail); x axis: time; color: local curvature (1/px). (I) Larva has minimal curvature variation. (J) Juvenile: increased mid-body curvature dynamics. (K) Adult: sustained high curvature in posterior region, reflecting increased amplitude and curvature of tail-beating. (L–S) Developmental comparison of behavioral metrics (individual animals shown as points). Boxplots: median (center line), interquartile range (box), 1.5× IQR (whiskers). Kruskal-Wallis tests with Holm-corrected post-hoc Mann-Whitney U tests: ∗*p* <0.05, ∗∗*p* <0.01, ∗∗∗*p* <0.001, ∗∗∗∗*p* <0.0001 (L). Coverage anisotropy (dimensionless): larvae exhibit minimal directionality; juveniles and adults show similar moderate anisotropy. (M) Thigmotaxis (fraction of time near boundaries): larvae show minimal boundary preference; adults display strongest thigmotaxis. (N) Velocity (μm/s): adults display 10-fold higher velocities than larvae/juveniles (larvae median = 86 μm/s, juvenile = 342 μm/s, and adult = 2,847 μm/s). (O) Absolute angular velocity (deg/s): progressive increase across development (larva = 21 deg/s, juvenile = 38 deg/s, and adult = 102 deg/s). (P) Wave index (dimensionless metric of undulatory motion): adults show reduced wave propagation compared to juveniles, consistent with localized tail-beating. (Q) Tail-beat amplitude (px): adults exhibit 5-fold larger amplitudes than earlier stages. (R) Tail curvature (1/px): adults display dramatically increased curvature compared to larvae/juveniles. (S) Tortuosity (path length/displacement, dimensionless): adults show highest path complexity (median = 3.2) compared to larvae (1.8) and juveniles (2.1). For all panels larvae *n* = 159; juveniles *n* = 106 and adults *n* = 60. Please also see Figures S1 and S2; Table S1; Videos S1 and S2.

The appendicularian *Oikopleura dioica* develops in an extremely rapid manner, attaining a motile larval stage within ∼7 h postfertilization (Figure 1B). This stage is succeeded by a juvenile stage, just prior to which the tail undergoes a remarkable 90° counterclockwise rotation relative to the trunk^21^^,^^27^^,^^28^ (Figure 1B). This rotation reflects the direction of the tail beat. In contrast to ascidians, cephalochordates and lower vertebrates whose tails beat in a left-right direction, *Oikopleura* juvenile tail beats in a dorsal-ventral direction.^21^ One day after fertilization *Oikopleura* matures to the adult form and lives for 7 days at 12°C (Figure 1B). After metamorphosis to juveniles, individuals produce a complex mucus structure known as the house.^29^ This is a structure that is actively secreted by a specialized trunk epithelium and then inflated around the animal which, through a controlled sequence of behavioral movements of the animal itself, employs the house for filter feeding.^30^^,^^31^^,^^32^ Once the house becomes clogged with particles, filtration efficiency drops. *Oikopleura* then abandons the old house and rapidly inflates a new house. The house is replaced multiple times each day (∼every 3 to 4 h).^33^^,^^34^

*Oikopleura* presents itself as an appealing organism to study behavioral change across ontogeny in a tunicate whose swimming is not abolished by metamorphosis^35^ for a number of reasons. The development of the *Oikopleura* nervous system across ontogeny is well characterized. Most neuronal cell divisions are complete by the onset of the juvenile stage and cell numbers change only modestly thereafter (60–78 neurons in the brain ganglion and stable in the nerve cord, specifically 25–30 neurons in the caudal ganglion and 30 in the caudal nerve cord).^12^^,^^13^^,^^14^ Muscle innervation begins at the larval stage, while more complex innervation of muscles has been documented at the adult stage.^12^ Interestingly, neurotransmitter expression changes dynamically across *Oikopleura* ontogeny. In larvae, the cholinergic marker vesicular acetylcholine transporter (VAChT) is restricted to three putative motoneurons, whereas in juveniles and adults the number of VAChT-positive neurons gradually increases to approximately twenty, likely corresponding to an expanded motoneuron population^15^ (Figure S1). Similarly, a small group of GABAergic neurons is present in the first brain nerves and the cerebral ganglion at the larval stage, with additional GABA-positive neurons appearing during juvenile and adult stages in these regions, as well as in the caudal ganglion^14^ (Figure S1).

Tail movements arise as brief myogenic twitches in post-hatching larvae and consolidate into coordinated tailbeats by the end of the larval stage.^32^ Recent studies have begun to describe *Oikopleura* behaviors related to house building (e.g., nodding) and house function in feeding.^30^^,^^31^^,^^32^^,^^36^^,^^37^

Motivated by the modest post-embryonic changes in nervous system organisation^12^^,^^13^^,^^14^^,^^15^ and studies in other animals,^7^^,^^11^^,^^38^^,^^39^ we hypothesize that behavioral development in *Oikopleura dioica* primarily involves changes in the deployment of existing motor patterns rather than the appearance of novel behaviors.

We further predict that developmental changes in swimming behavior would be described by shifts in the contribution and combination of a small set of postural modes. This expectation derives from previous work showing that locomotor behavior in diverse taxa can be described by a small number of low-dimensional postural modes whose relative usage changes across contexts and developmental stages.^18^^,^^38^^,^^40^^,^^41^^,^^42^ Finally, because the *Oikopleura* house imposes substantial mechanical and functional constraints on locomotion and is specialized for filter feeding,^29^^,^^30^^,^^31^^,^^32^^,^^33^ we hypothesize that house occupancy will restructure behavioral dynamics relative to free-swimming adults outside the house.

Here we built a behavioral atlas of *Oikopleura dioica* across its life cycle using high-throughput tracking and unsupervised (machine-learning based) analysis. We found that kinematic parameters increased during development, as did arena exploration. Kinematics and arena exploration differed markedly depending on whether *Oikopleura* was in-house (filter-feeding) or out-of-house (the period that the animal is free-swimming before inflating a new house). By employing dimensionality reduction, we derived low-dimensional representations of body postures, which we termed “*eigenoikopleuras*.” With these, we can describe the majority of postural variance in the *Oikopleura* larvae, juveniles, and adults. Leveraging a stage-balanced t-SNE (t-distributed stochastic neighbor embedding) space, we quantified behavioral dynamics across development, identifying a core set of clusters conserved across development as well as clusters that are stage-specific. Similarly, a small subset of clusters distinguishes between in-house and outside-house behaviors. Finally, we modeled *Oikopleura* behavior using a hidden Markov model (HMM) revealing the gradual temporal refinement in behavioral output rather than abrupt reorganization.

## Results

### Developmental changes in *Oikopleura dioica* locomotor behavior

To study behavioral development in the appendicularian *Oikopleura dioica* we recorded videos of larvae, juveniles, and adults (in-house or out-of-house) (Videos S1, S2, S3, and S4) in our tracking setups^18^^,^^43^ and used DeepLabCut^44^ to extract, in a high-throughput manner positional coordinates of multiple body parts of each *Oikopleura* larva, juvenile, and adult. Center of mass trajectories suggested that larvae exhibited simple episodic swimming with few changes in direction of travel (Figure 1C). Juveniles exhibited more complex swimming patterns with local runs and turns, yet they explored only a fraction of the arena (Figure 1D). Finally, adults exhibited very complex trajectories exploring most of the arena (Figure 1E).

Video S2. Video showing *Oikopleura dioica* juveniles swimming in the behavioral arena, related to Figure 1

Video S3. Video showing an *Oikopleura dioica* adult freely-swimming in the behavioral arena, related to Figure 2

Video S4. Video showing an Oikopleura dioica adult in-house filter feeding The animal is located inside the behavioral arena (related to Figure 2)

Larvae and juveniles were characterized by straight midlines which showed small deflections to the right and the left potentially indicating that simple swinging of the tail was sufficient for the animals to move (Figures 1F, 1G, 1I, and 1J). By contrast adults were marked by sigmoidal midline skeletons, showing increased tail amplitude and curvature and more waves traveling along their tail compared with larvae and juveniles (Figures 1H–1K).

In line with the changes in swimming and exploratory characteristics, this visual difference in the extent to which they explored the arena was quantified through the arena coverage anisotropy with the larvae showing the highest anisotropy (i.e., only exploring small regions of the arena) and adults exhibiting much lower anisotropy values indicative of exploring the arena in a more thorough and uniform manner (Figure 1L; Table S1).

Adults displayed a significantly higher thigmotaxis compared to larvae and juveniles (Figure 1M; Table S1). We have previously shown that *Ciona intestinalis* larvae are also thigmotactic^43^ suggesting that thigmotaxis may be an ancestral feature of chordate behavior, which may have evolved independently in arthropods.^45^^,^^46^

In addition, behavioral traits such as velocity, angular velocity, tail amplitude, tail curvature, and wave index increased progressively across developmental stages, with the most significant increase between the juvenile to the adult stages (Figures 1N–1R; Table S1). We also quantified the tortuosity (complexity of a trajectory) of the animal trajectories. We found that tortuosity significantly increased during juvenile and adult stages (Figure 1S; Table S1), suggesting it could be an adaptive behavioral trait. Because body size increases substantially between developmental stages (Figures 1B and S2A; Table S2), we asked whether the observed differences in motor parameters could be explained by changes in body size. After normalizing kinematic variables by body size, including speed, acceleration, and tail-beat amplitude, stage-specific differences in motor features persisted (Figures S2B–S2D; Table S2). Moreover, curvature-based and spatial measures, such as trajectory tortuosity and coverage anisotropy, are independent of body length and are therefore unlikely to be confounded by changes in body size.

### Adult behavior inside versus out-of-house

*Oikopleura* maintains efficient filter-feeding capacity by a house that surrounds the animal (Figure 2A), which is secreted from the trunk epithelium in the free-swimming animal (Figure 2B) and is inflated within an average of 21 seconds by a sequence of intricate motions (Figure S3). We asked how the presence of the house modified the behavior of adult *Oikopleura*. We found that in-house *Oikopleura* adults displayed limited exploration of the arena in contrast to the animals swimming out-of-house (Figures 2C, 2D, S4A and S4B). In line with this, pronounced differences were observed in tail swinging patterns and curvature between in-house to that of out-of-house phenotypes (Figures 2E–2H). Animals found out-of-house had significantly reduced anisotropy and were more thigmotactic compared to animals in-house (Figures 2I and 2J; Table S3). We then compared the kinematics of tail movement inside versus out-of-house. Compared to in-house animals, we observed animals out-of-house had significantly lower tail beating symmetry, wave index, and tail curvature in addition to increased tail beating amplitudes (Figures 2K–2N; Table S3). Despite showing limited overall displacement while in-house, *Oikopleura* moves its tail, creating a current that draws water through the house’s filters to allow the animal to feed.^30^^,^^33^ Tail beating in-house shows greater lateral bias, suggesting that *Oikopleura* adults in-house consistently move their tail more frequently to one side. Tail movements in-house are characterized by a higher wave index (i.e., the tail has higher curvature but lower tail-beat amplitude). The difference may reflect a behavioral adaptation for facilitating feeding while in-house. By contrast, the symmetric higher amplitude tail beating of free-swimming adults is more suited for straight trajectory swimming out-of-house.

**Figure 2:**
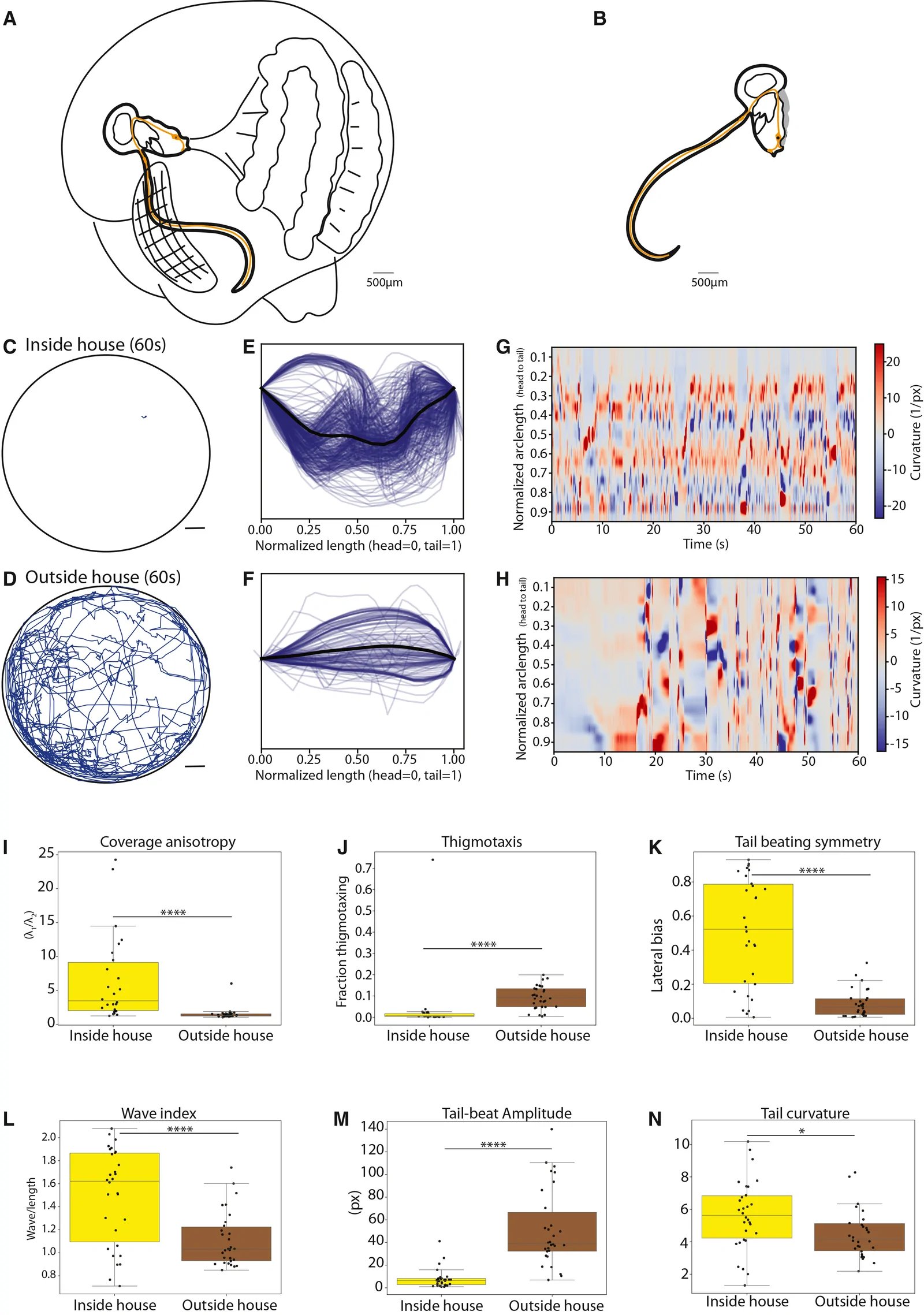
Adult behavior inside versus outside house (A) Schematic of adult *Oikopleura* within-house structure and free-swimming outside house (B). (C–D) Representative 60 s trajectory traces. (C) In-house: minimal spatial displacement. (D) Outside house: extensive spatial exploration within-house boundaries. For both panels the scale bars correspond to 1 mm. (E) Normalized centerline overlay in-house (black = individual, dark blue = mean). (F) Normalized centerline overlay outside house. (G–H) spatiotemporal curvature dynamics over 60 s. (G) Inside and (H) out-of-house. (I–N) Behavioral comparison between contexts (individual animals shown as points). Statistics: Mann-Whitney U tests with Holm correction: ∗*p* < 0.05, ∗∗∗∗*p* < 0.0001. Boxplots as in Figure 1. (I) Coverage anisotropy; (J) Thigmotaxis; (K) Tail-beating symmetry (lateral bias, dimensionless); Inside-house animals display strongly biased tail-beating. (L) Wave index (number of wave cycles per skeleton). (M) Tail-beat amplitude. (N) Tail curvature. For each condition (inside house and outside house) 30 animals were used in the analysis. Please also see Figures S3 and S4; Table S3; Videos S3, S4, and S5.

Overall, our data suggest that *Oikopleura* adults show significant ontogenic maturation in their locomotion kinematics and arena exploration depending on whether they are in-house or they have escaped the house and are free-swimming. These results reveal that inside-house animals exhibit restrained, asymmetric movements optimized for filter-feeding, while outside-house animals engage in large-amplitude, symmetric propulsion for navigation.

### Principal component analysis reveals behavioral mode structure

Features like tail curvature, tail beating symmetry, velocity, and others can describe *Oikopleura* postures accurately, however, the richness in these descriptions comes at the cost of a very high dimensionality. We sought to obtain a simpler representation that would describe the broad range of postures that *Oikopleura* can obtain (as represented by a randomly sampled set of curvature values) without losing significant information.^18^^,^^40^^,^^41^ We performed a principal component analysis (PCA) and found that 95% of the observed variance in the curvature data can be explained with 7 eigenvectors (here after called *eigenoikopleuras*) (Figure 3A). Eigenvectors are low- dimensional representations of body postures that are used extensively in animal behavior literature to quantitatively characterize animal movement in a diversity of species including *Caenorhabditis elegans*, *Danio rerio*, *Drosophila melanogaster*, and *Ciona intestinalis.*^18^^,^^38^^,^^39^^,^^41^^,^^42^^,^^47^^,^^48^^,^^49^^,^^50^^,^^51^^,^^52^ Each frame in the videos recorded across all three stages can be approximated as a linear combination of the first seven principal components (PC1–PC7), i.e., the *eigenoikopleuras*. PC1 captures overall body bending, PC2 reflects an anterior-posterior curvature gradient, and PCs 3–7 encode higher-order shape modes and local curvature features (Figures 3B and 3C).

**Figure 3:**
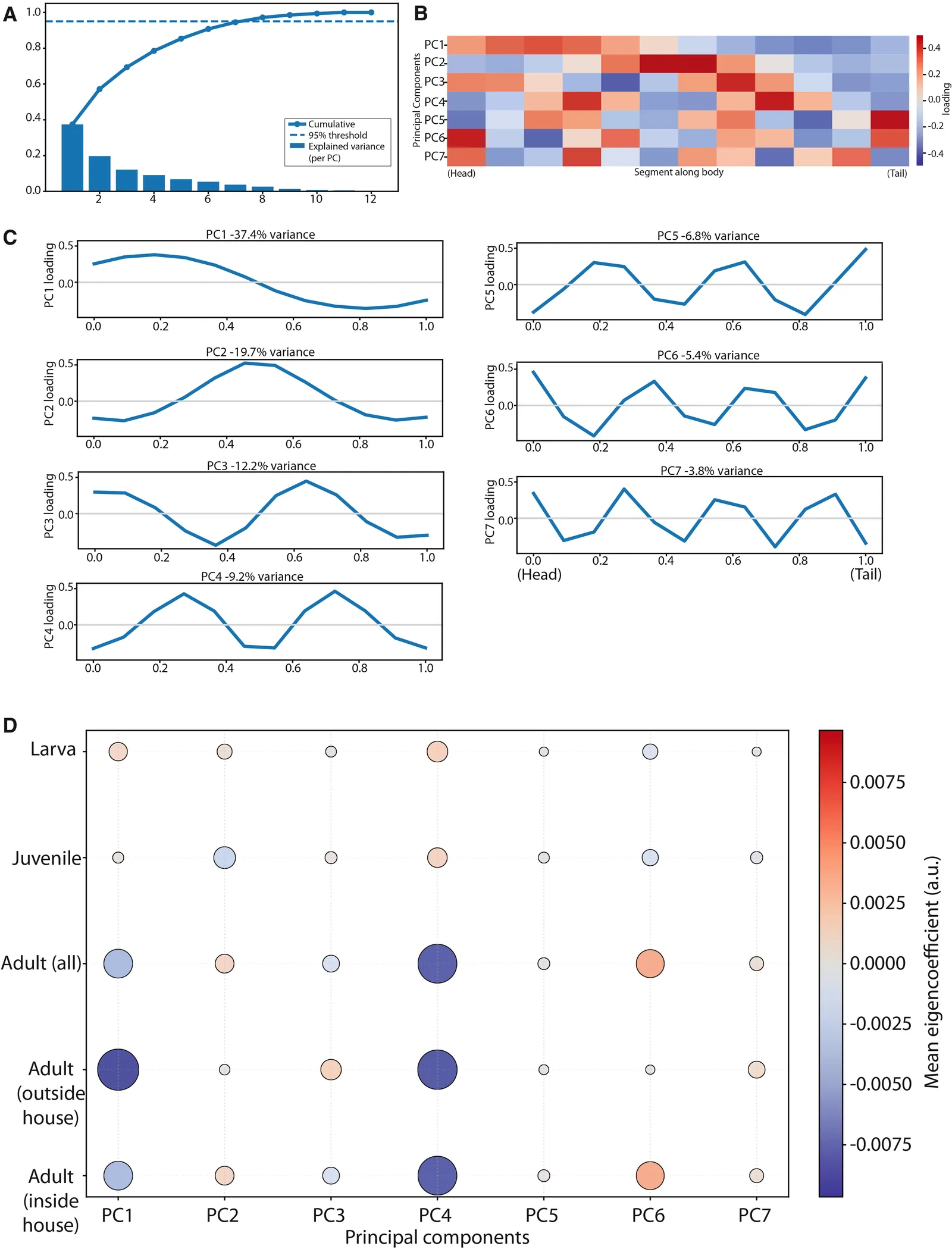
Principal component analysis reveals behavioral mode structure (A) PCA variance decomposition. Bar plot: variance explained per PC. Line: cumulative variance 7 PCs explain 95.3% total variance). Dashed line: 95% threshold. Seven PCs were retained for subsequent analyses (see STAR Methods). (B) PC loadings heatmap along body axis. Rows: PC1–PC7; columns: 21 evenly spaced points from head (left) to tail (right). Color: loading magnitude (red = positive, blue = negative). (C) PC1–PC4 spatial representations along normalized body length (0 = head, 1 = tail). Each plot shows PC loading magnitude across body axis, capturing progressively finer spatial modes related to tail dynamics and local curvature patterns. (D) Mean PC eigencoefficients by developmental stage and adult condition. Bubble size: mean coefficient magnitude; color: sign (red = positive, blue = negative). For the analysis presented in this figure we used 159 larvae, 100 juveniles, and 60 adults (30 inside the house and 30 outside the house). Please see Table S4 for further statistical information related to this figure.

To examine the difference in *eigenoikopleura* coefficient features, we used a bubble grid chart, where the hue represents the sign (i.e., positive or negative coefficient) and radius represents the mean amplitude (Figure 3D; Table S4). Thus, development reweights the same set of postural modes, with adults showing the broadest and most context-dependent PC usage. This analysis demonstrates that developmental stages and adult behavioral contexts occupy distinct regions of posture space, with adults exhibiting the greatest postural diversity.

### Unsupervised clustering reveals conserved behavioral modes across development

To probe how these postural modes assemble into behaviors we employed a low-dimensional spatiotemporal embedding approach to reveal stereotyped behaviors exhibited by *Oikopleura* across post-embryonic development. For this approach, we used as input 49656 frames from a subset of balanced data across all three developmental stages (i.e., 16552 frames per stage). Unsupervised embedding resolved seven recurring clusters of behavior (Figure 4A) spanning quiescent to burst-like dynamics. All stages are represented in the single behavior space, but cluster occupancy is developmentally biased: larvae and juveniles emphasize lower-intensity regimes (clusters 1, 3, and 4) (Figures 4B, 4C, S5A–S5D, and S6; Table S5) whereas adults disproportionately populate high-performance states (clusters 0 and 6) (Figures 4D, S5E–S5H, and S6; Table S5). In adults, context (in-house vs. out-of-house) further reweights state usage. For example, clusters 0 and 4 are overrepresented in in-house adults (relative to out-of-house adults), while clusters 1, 2, and 5 are underrepresented (Figures 4E, S5E–S5H, and S6; Table S5). Only cluster 6 seems to be a novel state present in out-of-house adults but not in in-house adults (or any other developmental stage) (Figures 4E, 4F, and S5E–S5H, Video S5). Overall, our analysis indicates repertoire conservation with developmental and contextual reweighting.

**Figure 4:**
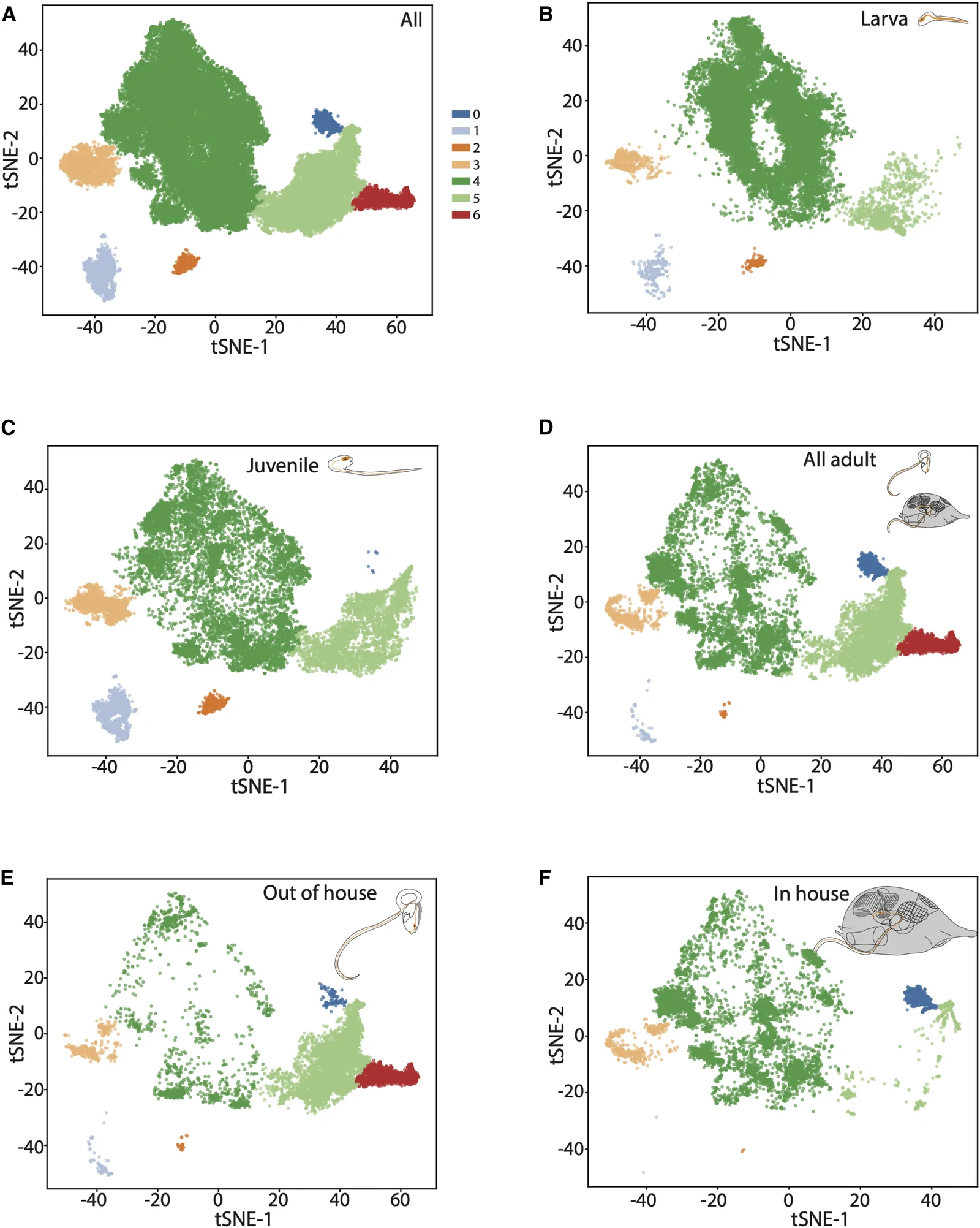
Unsupervised clustering reveals conserved behavioral modes across development (A) t-SNE embedding of behavioral windows (*n* = 45,872 windows from 57 individuals; 3 s windows, 1 s stride). Each point represents a behavioral window characterized by 8 features (velocity, acceleration, angular velocity, tail-beat frequency/amplitude, tortuosity, path complexity, and MSD slope; see STAR Methods). Dimensionality reduction: features 7 PCs (95.3% variance), t-SNE (perplexity = 750, stage-balanced training: *n* = 16552 per stage). Colors indicate HDBSCAN cluster assignment (7 states; parameters: min_cluster_size = 25, min_samples = 5, epsilon = 0.15×median distance). Gray: unassigned noise points (2.3% of windows). (B–F) Stage-specific embeddings showing behavioral state distributions. Please see also Figures S5 and S6; Table S6.

Video S5. Adult *Oikopleura dioica* in-house Cluster 6 spatiotemporal embedding, related to Figure 2

### Hidden Markov modeling reveals temporal structure of behavioral sequences

To move from cluster identity to temporal structure, we fit Gaussian HMMs (full covariance; K by BIC [Bayesian Information Criterion]) on the PC1–PC7 posture features. The emissions occupy distinct combinations of *eigenoikopleuras* (Figures 5A and S7A–S7H), and the resulting 13 hidden states provide a time-resolved scaffold over the seven unsupervised clusters.

**Figure 5:**
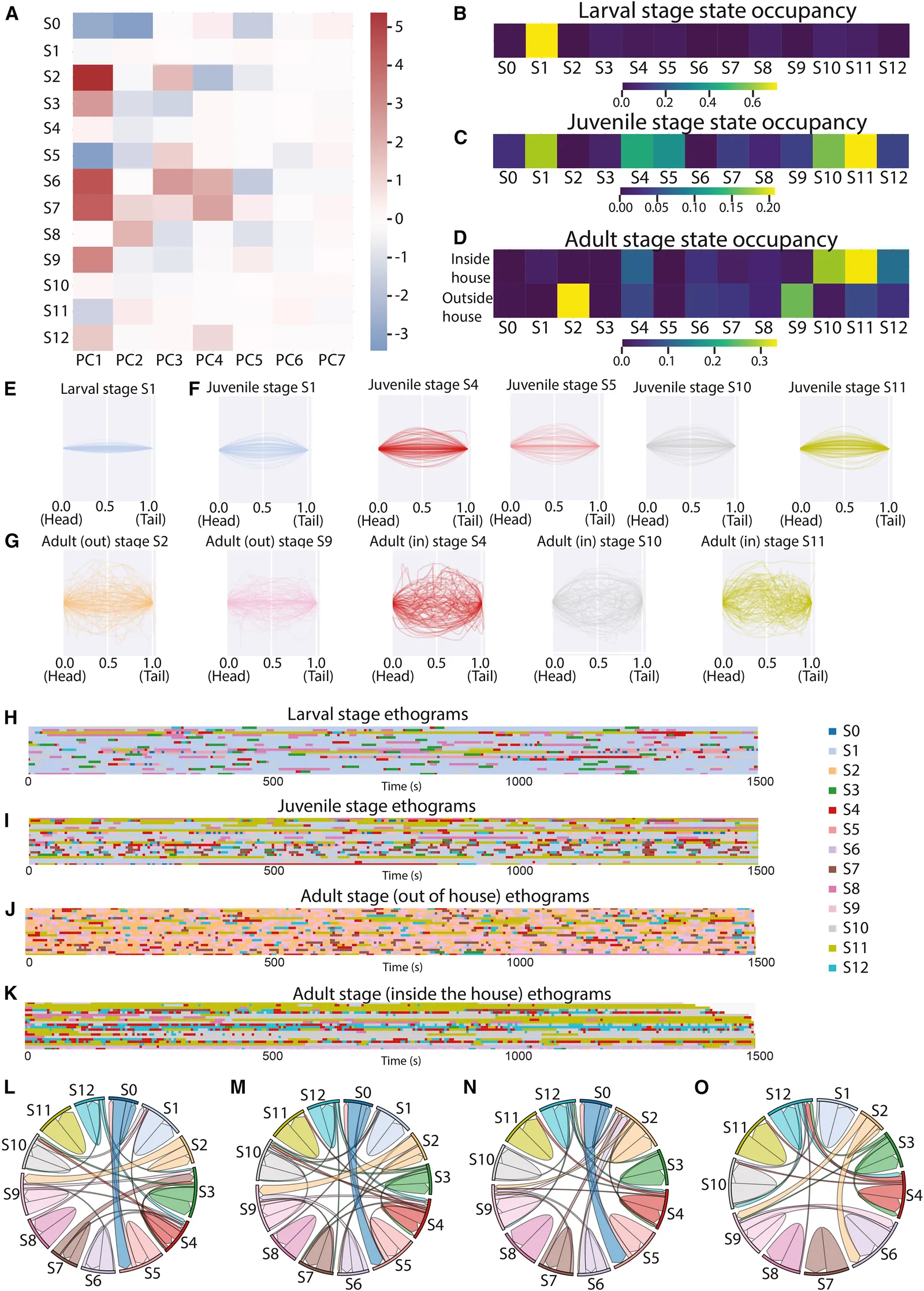
Hidden Markov model reveals temporal structure of behavioral sequences (A) HMM emission means in PC space. Heatmap shows mean PC1–PC7 values for each of 13 hidden states (S0–S12) identified by Gaussian HMM (full covariance; K selected by BIC; see STAR Methods). Rows indicate states; columns indicate PCs. Color indicates *Z*-scored PC value (red = high, blue = low). States capture distinct combinations of postural modes. (B–D) State occupancy (fraction of frames) by developmental stage and adult context condition. Color intensity indicates occupancy fraction. Each row sums to 1.0. (B) Larvae are dominated by states S0 (extended posture, 35% occupancy) and S1 (subtle undulation, 28%) with minimal occupancy of high-curvature states (S4, S11). (C) Juvenile stage. More distributed occupancy: S4 (moderate swimming, 21%), S5 (active tail-beating, 18%), with reduced S0 occupancy (15%) compared to larvae. (D) Adult stage occupancy by context. In-house: S4 (32%), S10 (18%), and S11 (15%) predominate states characterized by constrained, localized motion. Outside house: S2 (28%), S4 (22%), and S9 (16%) states predominate, consistent with extended postures and propulsion. (E–G) Representative state-specific body shapes. Normalized centerlines (*n* = 200 per state; gray = individuals, colored = mean) illustrate postural signatures. (E) Larval state S1 shows extended, minimal curvature while juvenile S1 show slight anterior bend. (F) Juvenile states: S4 (active swimming with moderate tail bend), S5 (pronounced undulation), S10 (localized tail curvature), and S11 (high-frequency tail motion with straight body). (G) Adult states: S2 (outside house, extended C-shape), S9 (outside, relaxed posture), S4 (inside, compact with tail wrap), S10 (inside, mid-body bend), and S11 (inside, tight posterior coil). (H–K) Ethograms showing HMM state sequences during 1,500 s sessions. Each row: one individual; colors: states S0–S12. (H) Larvae show prolonged state persistence with median dwell time S0 = 124 s, S1 = 89 s, reflecting limited behavioral repertoire. (I) Juvenile stage. Increased state diversity and transitions (median dwell times: S4 = 38 s, S5 = 29 s). (J) Adult outside house. Rapid state switching (median dwell S2 = 18 s) with episodic bursts of high-activity states. (K) Adult in-house. Complex temporal patterning with frequent transitions between S4, S10, and S11 (median dwell times: 22, 31, and 27, respectively), reflecting house-maintenance behaviors. (L–O) State transition diagrams (chord plots). Arc thickness: transition probability; color: originating state. (L) Larval stage transitions. Strong self-transitions with limited interstate switching. (M) Juvenile stage transitions. Reduced self-persistence increased cross-state transitions. (N) Adult outside house. Highly connected network with S2–S4, S4–S9, and S9–S2 as dominant transitions. (O) Adult in-house. Distinct transition structure: S4–S10 and S10–S11 form dominant cycles, reflecting stereotyped house-related behavior sequences. Model evaluation was performed on held-out test set comprising 20% of sequences: training log likelihood = -89,342; test log likelihood = -22,105; BIC = 179,284. Rare state pruning merged 1 state (<1% occupancy) into its nearest neighbor (see STAR Methods). Final model: K = 13 states, median state dwell time = 27 s (range: 8–124 s across stages/contexts). For the analysis presented in this figure we used 159 larvae, 100 juveniles, and 60 adults (30 inside the house and 30 outside the house). Please see also Figures S7–S9; Tables S6 and S7; Videos S6, S7, S8, S9, S10, S11, S12, S13, S14, S15, and S16.

State usage is developmentally and contextually reweighted (Figures 5B–5D, S7A–S7L, S8A–S8D, and S9; Tables S6 and S7): larvae concentrate in low-activity states (Figures S1, S9A, and S9B; Video S6; Table S7), juveniles distribute occupancy toward low-activity and moderate swimming (Figures S4, S5, S9A, S10, and S11; Table S7; Videos S7, S8, S9, S10, and S11), and adults shift with context, out-of-house favoring extended/propulsive states (like states; Figures S2, S6, S7, and S9A; Table S7; Videos S12 and S13) and in-house favoring constrained, localized motion (as states S4, S10, and S11; Figure S9A; Table S7; Videos S14, S15, and S16). States S4, S10, and S11 are shared across stages but with context-dependent skeleton shapes, suggesting behavioral homology despite morphological differences. Representative centerlines confirm distinct postural signatures (Figures 5E–5G and S7I–S7L).

Video S6. Larva *Oikopleura dioica* behavior corresponding to HMM state S1, related to Figure 5

Video S7. Juvenile *Oikopleura dioica* behavior corresponding to HMM state S1, related to Figure 5

Video S8. Juvenile *Oikopleura dioica* behavior corresponding to HMM state S4, related to Figure 5

Video S9. Juvenile *Oikopleura dioica* behavior corresponding to HMM state S5, related to Figure 5

Video S10. Juvenile *Oikopleura dioica* behavior corresponding to HMM state S10, related to Figure 5

Video S11. Juvenile *Oikopleura dioica* behavior corresponding to HMM state S11, related to Figure 5

Video S12. Adult (out of house) *Oikopleura dioica* behavior corresponding to HMM state S2, related to Figure 5

Video S13. Adult (out of house) *Oikopleura dioica* behavior corresponding to HMM state S9, related to Figure 5

Video S14. Adult (in house) *Oikopleura dioica* behavior corresponding to HMM state S4, related to Figure 5

Video S15. Adult (in house) *Oikopleura dioica* behavior corresponding to HMM state S10, related to Figure 5

Video S16. Adult (in house) *Oikopleura dioica* behavior corresponding to HMM state S11, related to Figure 5

The HMM also exposes sequencing (Figures 5H–5K): larvae show long dwell times and strong self-persistence; juveniles break into more frequent transitions; adults exhibit rapid switching out-of-house and stereotyped maintenance cycles inside (S4-S10-S11). Transition graphs formalize these patterns (Figures 5L–5O), with S4/S10/S11 acting as hubs. A prevalent transition in three out of the four conditions explored was the S0 to S5. Inspection of the ethograms reveals that long stretches of behavioral activity dominated by S5 cluster are interrupted by short periods of S0. This transition

may reflect a need for a short-term increase in speed during the moderate speed swimming phases of the *Oikopleura* ontogeny (Figures 5L and 5M). Clusters S0 and S5 are absent when the adult *Oikopleura* are feeding in-house, suggesting that being in-house limits the range of movements/behaviors that the adults can carry out or that these clusters are not compatible with filter-feeding behavior (Figure 5O). Therefore, the transition between S0 and S5 clusters is also absent (Figure 5O). Together, these results show that development and adult context reshape how conserved behaviors are chained in time-altering dwell times and transition backbones-rather than introducing entirely new states.

## Discussion

Using high-throughput tracking and multidimensional behavioral analysis, we characterize how the behavioral repertoire of *Oikopleura dioica* changes across ontogeny. By embedding behavioral data from different developmental stages and contexts into a shared low-dimensional postural framework, we show that behavioral maturation is driven not by the emergence of new motor acts, but by systematic changes in posture occupancy, kinematics, exploration, and state-transition dynamics within a conserved motor repertoire. Behaviors form structured regions of a common postural manifold, a map of the animal’s possible body postures and the transitions between them, and increasing behavioral complexity arises through the progressive reweighting, sequencing and modulation of conserved motor primitives across development.

### Behavioral development in a comparative chordate and metazoan context

Comparative chordate studies suggest a conserved developmental progression of locomotor maturation, from rudimentary movements to coordinated adult swimming.^53^ In amphioxus (*Branchiostoma floridae*), this transition involves increasing coordination and propulsive efficiency as locomotion shifts from ciliary to muscle-driven swimming.^54^ Correspondingly, *Oikopleura* larvae and juveniles rely on whole-body bends, whereas adults exhibit sustained undulatory swimming.

Similar developmental increases in locomotor activity and thigmotaxis (an adaptive behavior linked to predator avoidance^55^^,^^56^^,^^57^^,^^58^) have been reported in zebrafish and amazon mollies,^11^^,^^59^ and age-dependent shifts in behavioral composition occur in insects such as *Drosophila*^39^ and honeybees.^60^ Together, these parallels suggest that behavioral maturation may follow conserved principles across diverse animal lineages.

As a permanently pelagic tunicate that retains active tail-driven locomotion throughout its life cycle, *Oikopleura* uniquely enables comparisons of locomotor behavior throughout development, unlike *Ciona*, which shows improved swimming performance confined to larval stage before metamorphosis,^43^ Consistent with age-dependent behavioral changes in *Ciona*, *Oikopleura* undergoes progressive refinement of locomotor abilities that may enhance dispersal and habitat navigation.

### Revisiting classical behavioral categories through a postural manifold framework

Previous work based on video analysis identified discrete tail movement categories in *Oikopleura dioica*, bending, swimming, nodding, and filtering that emerge along distinct developmental timelines.^32^ Bending and swimming appear shortly after hatching and are progressively refined, whereas nodding and filtering emerge only in young adults coincident with first house production.^32^ Our results are consistent with this developmental sequence but extend it in several important ways. Notably, several *eigenoikopleuras* align closely with tail shapes previously extracted by Kreneisz and Glover,^32^ bending, swimming, and nodding map primarily onto *eigenoikopleuras* 1–4 (PC1–PC4), whereas filtering corresponds mainly to *eigenoikopleuras* 5–7 (PC5–PC7). In addition, increases in wave index, tail-beat amplitude, velocity, and behavioral transition rates observed here closely parallel the refinement of tail kinematics described previously.^32^

These findings reframe classical movement categories as stage- and context-biased expressions of a shared motor repertoire. House-associated behaviors such as nodding and filtering, previously treated as adult-specific actions, emerge as constrained and stereotyped reconfigurations of common motor modules optimized for filtration in-house. Thus, behavioral complexity in *Oikopleura* arises through progressive reweighting, sequencing, and modulation of conserved motor primitives across ontogeny.

### Potential neural and neuromuscular mechanisms underlying behavioral maturation

The molecular and neural mechanisms underlying behavioral plasticity in *Oikopleura* remain poorly understood. In many species, developmental changes in locomotion arise from context-dependent sensory inputs, neuromodulation, and maturation of neuromuscular interfaces rather than extensive circuit rewiring.^61^^,^^62^^,^^63^ In *Oikopleura*, neurotransmitter systems are likely contributors to these behavioral transitions, as the number and distribution of cholinergic and GABAergic neurons increase from larval to adult stages despite largely stable overall neuron numbers.^14^^,^^15^^,^^64^ Pharmacological manipulation of cholinergic and GABAergic signaling could reveal how swimming kinematics and posture are controlled across development. Comparing effects across larval, juvenile, and adult stages could reveal developmental shifts in neurotransmitter dependence and behavioral control.

Behavioral maturation also coincides with substantial neuromuscular development.^12^ Tail movements progress from brief myogenic twitches in larvae to coordinated tailbeats and sustained, high-amplitude swimming in juveniles and adults. Neuromuscular synapses mature progressively, reaching functional maturity around the juvenile stage and peaking in day 3 adults.^12^ This temporal correspondence suggests that strengthened neuromuscular coupling underlies the observed increases in tail curvature, beat amplitude, velocity, and state-transition dynamics. Thus, behavioral maturation reflects refinement of motor output within a compact nervous system.

### Putative contributions of sensory development and sensimotor integration to behavioral maturation

In parallel with neuromuscular maturation, sensory circuit development and sensory-motor integration are likely to become increasingly influential as locomotor competence improves. Mechanosensory systems such as Langerhans cells and the coronal organ connect to *Oikopleura*’s central nervous system early in development.^65^^,^^66^^,^^67^ Langerhans cells are secondary sensory cells which are putative mechanoreceptors (they may have additional unknown functions), which when activated elicit an escape response.^66^^,^^68^ Similarly, the coronal organ is composed of secondary sensory cells functioning as mechanoreceptors.^67^

As swimming becomes faster, stronger, and more flexible, sensory feedback may exert tighter control over exploration, thigmotaxis, and behavioral state selection, consistent with the increased behavioral diversity and faster state switching revealed by our HMM analyses. Together, these processes support a model in which complex behavior emerges through coordinated maturation of neuromuscular and sensory systems acting on a conserved motor repertoire.

Future studies can leverage established tools in *Oikopleura* and related tunicates (e.g., CRISPR-Cas9 genome editing,^69^ calcium imaging, and chemogenetics^70^^,^^71^^,^^72^^,^^73^) to uncover the roles of neurotransmitters and sensory cells on behavioral developmental across ontogeny and behavioral plasticity.

### Biomechanical constraints on behavioral ontogeny

Ontogenetic changes in morphology and biomechanics are likely to bias access to specific behavioral states. Developmental shifts in tail orientation, curvature, and amplitude,^74^ as well as changes in trunk geometry relative to tail length,^75^ alter the biomechanical context in which motor commands are executed.

For example, in larvae, the tail projects posteriorly and the trunk is relatively large and ovoid; in juveniles and adults, the tail shifts ventrally, forming an angle of ∼60° relative to the trunk axis.^74^ Behavioral development in *Oikopleura* therefore likely reflects an interaction between neural control and evolving biomechanical constraints that shape the energetic cost and stability/persistence of different motor states.

### Behavioral specialization associated with house occupancy

Significant differences in adult *Oikopleura* locomotion in-house versus out-of-house likely reflect two distinct behavioral states (see also study limitations section). In-house, tail movements primarily sustain filtration, because water flow through the house is needed for feeding, and houses are discarded once they become clogged with particles.^30^^,^^31^^,^^32^^,^^33^^,^^76^ In contrast, adults swim rapidly and in a more complex manner when out-of-house, a phase repeated several times daily as they discard and rebuild their houses.

This fast, directed swimming may help them move away from poor feeding conditions, avoid predators, or migrate vertically through the water column.^77^ The brief out-of-house intervals are the only stage when directional behaviors such as rheotaxis or thermotaxis could occur, although neither has been demonstrated in *Oikopleura*. This high-speed swimming phase may therefore provide an adaptive window for environmental assessment and habitat selection before commitment to a new filtration house.

### Ecological significance of developmental changes in exploration and thigmotaxis

Developmental changes in exploration and thigmotaxis likely reflect age-dependent trade-offs between predator avoidance and resource acquisition. Larvae exhibit low thigmotaxis, short swimming bouts, and predictable movements. In many taxa, this period of vulnerability is associated with either strong thigmotaxis or reduced motility.^78^^,^^79^^,^^80^^,^^81^^,^^82^ *Oikopleura* larvae may adopt the latter strategy: because they do not feed, reduced activity may minimize predation risk at little energetic cost. In contrast, juveniles and adults exhibit greater thigmotaxis, broader exploration, and less predictable trajectories. Staying near boundaries and moving unpredictably may reduce predation risk^83^ while supporting active foraging, marking a clear shift from the larval strategy.

Importantly, increased swimming competence and repeated house renewal enable exploration, resource acquisition, and habitat assessment, supporting growth and reproduction. By modulating swimming behavior, *Oikopleura* maintains efficient feeding across a range of food conditions, contributing to its success in both oligotrophic and eutrophic environments.^31^ Juvenile and adult *Oikopleura* pump water through their mucous houses to filter phytoplankton, helping transfer carbon from surface waters to the deep ocean. When a house becomes clogged, it is discarded and sinks rapidly, transporting organic matter to the seafloor where it provides an important food source for benthic communities.^30^^,^^76^^,^^84^^,^^85^^,^^86^^,^^87^

### Behavioral complexity in a miniaturized chordate nervous system

Despite possessing one of the smallest known chordate nervous systems (∼130 neurons^12^^,^^13^^,^^14^ in contrast, for example, to the zebrafish larval nervous system which is composed of ∼100,000 neurons^88^^,^^89^), *Oikopleura* exhibits a remarkably rich behavioral repertoire. Nervous system miniaturization has occurred independently across several lineages,^90^^,^^91^ including rotifers,^92^ tardigrades,^93^ nematodes,^94^ and other chordates,^95^ often in association with body size reduction or specialized lifestyles.^91^^,^^96^ While fewer and smaller neurons might be expected to limit sensory acuity or motor precision, examples such as *Oikopleura* demonstrate that miniaturized nervous systems can nonetheless support complex, adaptive behavior.^90^ This suggests that nervous system miniaturization reflects alternative evolutionary solutions in which behavioral performance is achieved through architectural and computational efficiency.

### Future directions: Environmental stress and behavioral plasticity

While we have characterized behavioral changes across *Oikopleura* ontogeny, an important open question is how early-life environmental stress shapes behavioral plasticity later in life.^97^ Marine species remain underrepresented in stress research, despite the sensitivity of *Oikopleura dioica* to anthropogenic stressors.^98^^,^^99^ Integrating ontogenetic behavioral tracking with controlled early-life perturbations, together with genetic and neurophysiological tools available in tunicates (e.g., CRISPR-Cas9, calcium imaging, and chemogenetics^70^^,^^71^^,^^72^^,^^73^^,^^100^), provides a powerful framework for dissecting how compact nervous systems generate flexible behavior across development.

### Limitations of the study

We find that *Oikopleura dioica* exhibits progressively increased thigmotaxis from larval to adult stages. A limitation of the present study is that we did not systematically examine how arena geometry influences thigmotactic behavior across developmental stages. Work in rodents has shown that while some quantitative thigmotaxis metrics can depend on arena shape, overall levels of motor activity and wall-directed behavior are often comparable across geometries.^101^^,^^102^ Nevertheless, testing multiple arena shapes in *Oikopleura* would strengthen the generality of our conclusions and help dissociate geometry-dependent effects from developmental changes in spatial behavior.

Our study uses a low-dimensional postural framework based on eigenvectors (*eigenoikopleuras*) to capture the majority of body-shape variance across ontogeny. A conceptual limitation is that we did not perform cross-species comparisons of eigenpostures. Such analyses would allow a rigorous test of whether observationally defined movement categories correspond to shared low-dimensional postural spaces across phylogenetically distant chordates, or whether eigen-based approaches reveal behavioral structure not captured by classical categorical descriptions. We view cross-species eigenposture comparisons as an important future direction for quantitative behavioral evolution across the chordate tree of life.

More broadly, deeper insight into the evolution of behavioral change across ontogeny in chordates will require examining a wider range of invertebrate chordates. In particular, amphioxus, owing to its phylogenetic position and comparatively larger and more complex nervous system, may provide a closer approximation to aspects of the ancestral chordate condition.^16^^,^^17^^,^^103^^,^^104^

A further technical limitation of our behavioral setup is the restricted vertical depth available to adult *Oikopleura*. This constraint may limit the expression or detection of behaviors that depend on vertical displacement or gravity sensing, such as directional vertical swimming. Imaging *Oikopleura* under conditions that approximate the natural water column is challenging; however, emerging approaches such as scale-free vertical tracking microscopy^105^ offer promising avenues for tracking *Oikopleura* and other zooplanktonic organisms under more ecologically realistic conditions.

Finally, a key limitation of our comparison between adult behavioral contexts is that the “inside-house” and “outside-house” conditions differ not only in behavioral context (feeding and house maintenance versus free swimming) but also in the physical environment experienced by the animal. The house imposes mechanical confinement, altered boundary geometry, and distinct hydrodynamic conditions, including flow fields generated during filtration. Any of these factors could, in principle, contribute to the observed differences in kinematics, posture usage, and behavioral state occupancy. Because of our experimental design, we cannot fully disentangle active, context-dependent behavioral regulation from passive biomechanical or fluid-dynamic constraints imposed by the house itself.

Future work could address this limitation by experimentally decoupling physical constraint from behavioral context, for example, by comparing free-swimming adults in artificial chambers that mimic house confinement without filtration flow, or by imposing controlled flow fields around free-swimming animals. Integrating quantitative behavior with direct hydrodynamic measurements or modeling would further clarify which aspects of the inside/outside differences reflect adaptive behavioral organization versus environmental constraint.

## Resource availability

### Lead contact

Requests for further information, resources, and reagents should be directed to and will be fulfilled by the lead contact, Marios Chatzigeorgiou (m.chatzigeorgiou@imperial.ac.uk).

### Materials availability

This study did not generate any new materials.

### Data and code availability

• Raw video data have been deposited in Zenodo: (https://doi.org/10.5281/zenodo.17502188, https://doi.org/10.5281/zenodo.17464322, https://doi.org/10.5281/zenodo.17455742, and https://doi.org/10.5281/zenodo.17503716).
• The DeepLabCut-based DNN which recognizes landmarks on *Oikopleura* larvae, juveniles, and adults and the training datasets have been deposited in Zenodo: (https://doi.org/10.5281/zenodo.17473173, https://doi.org/10.5281/zenodo.17459389, and https://doi.org/10.5281/zenodo.17491493).
• All original code required to analyze our behavioral data has been deposited in Zenodo: (https://doi.org/10.5281/zenodo.22657165).
• Any additional information required to reanalyze the data reported in this paper will be made available from the lead contact upon request.

## Acknowledgments

We would like to thank members of the Chatzigeorgiou lab for valuable feedback on the manuscript. We acknowledge funding from the Michael Sars Centre core budget (NFR grant 234817 “Sars International Centre for Marine Molecular Biology Research, 2013–2022”) and grants from the 10.13039/501100005416Research Council of Norway to M.C. (339399 and 335582) and D.C. (250005).

## Author contributions

Conceptualization, O.T., S.N., R.d.S.M.B., and M.C.; methodology, O.T., S.N., R.d.S.M.B., and M.C.; data curation, O.T., R.d.S.M.B., and S.N.; investigation, O.T., R.d.S.M.B., and S.N.; visualization, O.T., R.d.S.M.B., and S.N.; resources, M.C., D.C., and A.E.A; funding acquisition, M.C. and D.C.; project administration, M.C.; supervision, M.C.; writing – original draft, O.T., S.N., R.d.S.M.B., and M.C.; writing – review and editing, O.T., S.N., R.d.S.M.B., D.C., A.E.A., and M.C.

## Declaration of interests

The authors declare no competing interests.

## Declaration of generative AI and AI-assisted technologies in the manuscript preparation process

During the preparation of this manuscript, Microsoft Copilot was used for polishing and grammar checking of the manuscript text. All output text was critically reviewed and edited, and the authors take full responsibility for the final content.

## STAR★Methods

### Key resources table

**Table:** 

REAGENT or RESOURCE	SOURCE	IDENTIFIER
**Deposited data**		

Raw videos of *Oikopleura dioica* (larvae, juveniles, adults inside or out-of-house)	https://doi.org/10.5281/zenodo.17502188 https://doi.org/10.5281/zenodo.17464322 https://doi.org/10.5281/zenodo.17455742 https://doi.org/10.5281/zenodo.17503716	N/A
DLC DNN trained to recognize *Oikopleura dioica* body parts and training data	https://doi.org/10.5281/zenodo.17473173 https://doi.org/10.5281/zenodo.17459389 https://doi.org/10.5281/zenodo.17491493	N/A

**Software and algorithms**		

Code generated for Oikopleura behavioral analysis	https://doi.org/10.5281/zenodo.22657165	N/A
DeepLabCut	Lauer et al.^44^	N/A
Matplotlib	https://github.com/matplotlib/matplotlib	Version 3.10.8
Numpy	https://github.com/numpy/numpy	Version 2.3.5
scikit-learn	https://github.com/scikit-learn/scikit-learn	Version 1.5
SciPy	https://github.com/scipy/scipy	Version 1.16.3
pandas	https://github.com/pandas-dev/pandas	Version 2.3.3
Seaborn	https://github.com/mwaskom/seaborn	Version 0.13.2
openTSNE	https://github.com/pavlin-policar/openTSNE	Version 1.0.4
hmmlearn	https://github.com/hmmlearn/hmmlearn	Version 0.3.3
HDBSCAN	https://github.com/scikit-learn-contrib/hdbscan	Version 0.8.39

### Experimental model and study participant details

Wild *Oikopleura dioica* were collected in coastal waters near Bergen, Norway, and cultivated for several generations at the Michael Sars Center, University of Bergen, Norway, before use. The facility in which the animals were maintained was approved by the Norwegian Directorate of Health (Approval ID: 24_18878–2). Ethical approval was not required because *Oikopleura dioica* is not covered by the Norwegian Animal Welfare Act (§13). The animals were kept according to standard protocol at 12 ± 0.5°C resulting in a 7-day life cycle.^106^ Larvae (8 hpf), juveniles (18 hpf), and adults (120 hpf) were used in this study. Sex was not determined for any animals included in the analyses. At the larval and juvenile stages, sex cannot be identified morphologically, and individuals were therefore analyzed without regard to sex. Adult animals were not sexed prior to behavioral recording, precluding assessment of sex-specific differences in behavioral parameters.

### Method details

#### *Oikopleura dioica* behavioral recording

For the tracking of the *Oikopleura* in its different stages, we used an array of three behavioral setups which we have previously described.^18^ Key components include an IR-sensitive monochrome DMK 33UP1300 camera, fitted with an MVL75M1 lens and C-mount extension tubes (a CML10 and a CML25). For illumination, each behavioral setup was equipped with a PLA ring carrying infrared LEDs (peak emission 850 nm), providing dark-field illumination. Agarose arenas were fabricated using custom 3D-printed PLA molds.^18^ Briefly, 35-mm Petri dishes were filled with ∼9 mL of 0.8% agarose prepared in filtered sea water (FSW). While the agarose was still warm, the molds were inserted into the agarose and left in place until gelation. Molds were then removed, excess agarose was trimmed, and the arenas were hydrated with FSW. The resulting circular arenas measured 10 mm in diameter and 3 mm in depth, corresponding to an approximate volume of 240 mm^3^. The agarose coating of the arena is essential to prevent *Oikopleura* from sticking to the plastic Petri dish and also to diffuse uniformly the light coming in from the IR LEDs. The animals were recorded at 30 frames/second.

For the larval and juvenile stages, controlled fertilizations were performed. Eggs from naturally spawned females were fertilized with sperm pool obtained from a minimum of 3 to 4 males. The first cell division around 20–30 minutes after fertilization was checked to confirm the fertilization and the embryos were kept at 13°C until recording. For larval stage, fertilized eggs were incubated until 7–8 hours postfertilization (hpf) and larvae were recorded for a total of 10 minutes. For the juvenile stage, animals were incubated until 16–18 hpf and recorded for a total of 10 minutes.

For the adult stage, animals were recorded on day 5 for a total of 10 minutes. The first 5 minutes were recorded inside their houses (marked by the addition of *Synechococcus* cyanobacteria) and forced out of the house and recorded for additional 5 minutes to register both of their behavioral states (inside and outside their houses).

#### DeepLabCut model development for tracking of *Oikopleura dioica* in different developmental stages

To evaluate the behavior of the animals on the recordings obtained, three different models (larvae, juvenile and adult models) were developed using machine-learning software Deeplabcut (DLC) 3.0.0rc6, training the AI tool to detect and track the larvae, juvenile and adult animals. For the training of the models, 15–16 videos were manually annotated, with a minimum of 20 randomly selected frames per video.

#### Data processing

Behavioral tracking data were obtained using DeepLabCut (v3.0.0rc6) trained on manually annotated frames spanning all developmental stages (larva, juvenile, adult). Tracking confidence was filtered at likelihood ≥0.99, and statistical outliers were removed using the interquartile range method (IQR threshold = 2.0). Teleportation artifacts (frame-to-frame displacement >100 pixels) were eliminated. To ensure temporal continuity, only tracking segments ≥3 s (90 frames at 30 fps) was retained for larval and juvenile stages; adult stage segments required ≥1 s continuity. Internal gaps in tracking were filled using linear interpolation when bracketed by valid detections. Coordinates were temporally downsampled 1:5 (6 Hz) for computational efficiency while preserving behavioral dynamics.

#### Behavioral metrics extraction

Postural metrics: Body centrelines were extracted by interpolating through trunk and tail anchor points using piecewise cubic Hermite interpolation (PCHIP) to 21 evenly spaced points. Centrelines were normalized by translating the head to the origin, rotating the tail onto the positive x axis, and scaling total length to unity. Local curvature was computed using Savitzky-Golay filtering (window = 15 frames, polynomial order = 2) of centreline derivatives. Body axis orientation (theta) was defined as the angle from mean head position to mean tail position.

Kinematic metrics: Center of mass (COM) was calculated as the mean position of trunk markers. Instantaneous velocity was computed as the Euclidean displacement of COM between consecutive frames, converted to μm/s using a calibrated pixel-to-micrometer ratio (11.56 μm/pixel). Angular velocity (omega) was derived by unwrapping body axis angles within continuous tracking segments and applying finite differences, preventing NaN propagation across gaps. Acceleration was computed as the temporal derivative of velocity.

Tail-beat analysis: Tail-beat frequency was determined at native temporal resolution (30 fps) using fast Fourier transform (FFT) of the lateral tail displacement signal with Hann windowing. Dominant frequency was refined via quadratic interpolation of the peak spectral bin. Tail-beat amplitude was quantified as half the peak-to-peak displacement over 1 s windows.

Window-based features: Behavioral metrics were aggregated over sliding 3 s windows with 1 s stride. For each window, we computed: (1) 95th percentile velocity and acceleration, (2) fraction of frames above a movement threshold (defined as 3× median absolute deviation of per-individual velocity), (3) path tortuosity (path length / net displacement), (4) path complexity (entropy of normalized x-y correlation), and (5) mean-squared displacement (MSD) slope over short lags (1–3 frames).

#### Unsupervised behavioral state discovery

Feature preprocessing: Eight features were selected for behavioral state classification: tortuosity, path complexity, MSD slope, tail-beat frequency, tail-beat amplitude, velocity (95th percentile), acceleration (95th percentile), and angular velocity (95th percentile). Positive-valued features (velocity, acceleration, angular velocity, tail-beat amplitude) were log_1+_-transformed. Features were robustly clipped to the 0.5-99.5th percentile range and median-centered within individuals to account for inter-individual variability. Global standardization (*Z* score) was then applied across the dataset.

#### Dimensionality reduction and embedding

Preprocessed features were reduced to 7 principal components (PCs) using PCA, capturing >95% cumulative explained variance. To ensure balanced representation across developmental stages, data were downsampled such that each stage (larva, juvenile, adult) contributed equally to the training set (n = [minimum stage count] per stage). The balanced dataset was embedded in 2D using t-distributed stochastic neighbor embedding (t-SNE) via openTSNE with perplexity = 750 (affinity: approximate nearest neighbors; initialization: PCA; early exaggeration iterations = 250, total iterations = 1000).

Clustering: Behavioral states were identified using Hierarchical Density-Based Spatial Clustering of Applications with Noise (HDBSCAN). To determine optimal hyperparameters, we performed a grid search over min_cluster_size (15–30), min_samples (None, 1, 5), and epsilon (0–20% of median pairwise distance in embedding space), evaluating cluster stability and noise fraction. The resulting clustering yielded K = 7 distinct behavioral states. Cluster labels from the balanced embedding were propagated to the full dataset using k-nearest neighbors (k = 7, distance weighting) in the t-SNE embedding space.

#### Hidden Markov model analysis

Sequence preparation: Individual animal trajectories in PC space (7 dimensions) were treated as sequences, with minimum sequence length 30 frames. Sequences were split 80:20 into training and held-out test sets.

Model fitting. Gaussian Hidden Markov Models (HMMs) were fit with K = [5–15] hidden states using the hmmlearn package. For each K, models were initialized via K-means clustering of the training data to set emission means and covariances (full covariance matrices). Start probabilities were derived from cluster occupancy, and transition matrices were initialized with strong self-transition bias (0.8 diagonal). Expectation maximization (EM) was run for up to 500 iterations (tolerance = 10^-4^) from 8 random initializations, retaining the model with highest training log likelihood.

Model selection: Models were compared using Bayesian Information Criterion (BIC = -2·LL + p·log(N), where p = number of free parameters, N = total training frames) and held-out log likelihood. The selected model (K = 14) balanced parsimony and explanatory power.

State pruning: States occupying <1% of total frames were identified as rare and merged into the nearest state by Euclidean distance in emission mean space. The model was then refit with the reduced state count (K_final = 13).

Characterization: Viterbi decoding was applied to all sequences to infer the most likely state sequence. State dwell times (run lengths) were computed. State occupancy and transition probabilities were computed separately for each stage × experimental condition combination.

#### Shape analysis

For each behavioral state, normalized centerlines (n = [min 100, max 500] per state) were sampled from time windows assigned to that state. Quality filtering removed centerlines with lateral deviation >0.6 body lengths or adjacent segment angles >2.0 radians (∼115°). Mean centerline shapes were computed per state and visualized to reveal state-specific postural signatures.

### Quantification and statistical analysis

Analyses included 159 larvae (26.5 h of tracking), 106 juveniles (17.7 h), and 60 adults (10.0 h). After quality control, filtering, and resampling, the dataset comprised 572,559 frames for larvae, 381,706 frames for juveniles, and 216,060 frames for adults (1,170,325 frames in total; 54.2 h of behavior). A balanced subset of 49,656 frames (2.5 h) was used for clustering analyses. Behavioral metrics were averaged per individual across all time windows. Comparisons among the three developmental stages were performed using Kruskal-Wallis H-tests, followed by pairwise Mann-Whitney U tests with Holm-Bonferroni correction for multiple comparisons or Mann-Whitney U tests for comparisons between two groups. All statistical tests were two-tailed with the correction for multiple comparisons where applicable. Exact *p*-values are reported in the figure legends. Asterisks correspond as follows: ∗*p* <0.05, ∗∗*p* <0.01, ∗∗∗*p* <0.001, ∗∗∗∗*p* <0.0001. ns notation corresponds to *p* >0.05 Further details regarding the statistical tests, exact value of n (number of animals) are reported in the figure legends and in Tables S1, S2, S3, S4, S5, S6, and S7. Data presented in the form of boxplots show median (center line), interquartile range (box), 1.5× IQR (whiskers) unless otherwise stated in the figure legends.

## Supplemental information

Document S1. Figures S1–S9

Table S1. Statistical analysis of *Oikopleura dioica* kinematic and arena features, related to Figure 1

Table S2. Statistical analysis of differences in kinematic features across *Oikopleura dioica* ontogeny, related to Figure S2

Table S3. Statistical analysis of *Oikopleura dioica* kinematic and arena features, related to Figure 2

Table S4. Statistical analysis of *Oikopleura dioica* PC1–PC3, related to Figure 3

Table S5. Descriptive statistics of kinematic and spatial features underlying spatiotemporal embedding-derived clusters, related to Figures 4 and S6)

Table S6. Descriptive statistics for larval, juvenile and adult state occupancy, related to Figure 5

Table S7. Descriptive statistics of kinematic and spatial features underlying HMM states, related to Figures 5 and S9

Video S1. Video showing *Oikopleura dioica* larvae swimming in the behavioral arena, related to Figure 1
