# Supplementary material for: Behavioral ontogeny in a pelagic tunicate reveals developmental maturation and context-dependent modulation
Source: iScience. 2026 Nov 4;29(11):118104. doi: 10.1016/j.isci.2026.118104 (PMC13753599; doi:10.1016/j.isci.2026.118104)
Supplement: Supplementary file 1 [file mmc1.pdf]

iScience, Volume *29*

Supplemental information

Behavioral ontogeny in a pelagic tunicate reveals developmental maturation and context-dependent modulation

Oleg Tolstenkov, Rodolfo da Silva Mazzarini Baldinotti, Sissel Norland, Anne Elin Aasjord, Daniel Chourrout, and Marios Chatzigeorgiou

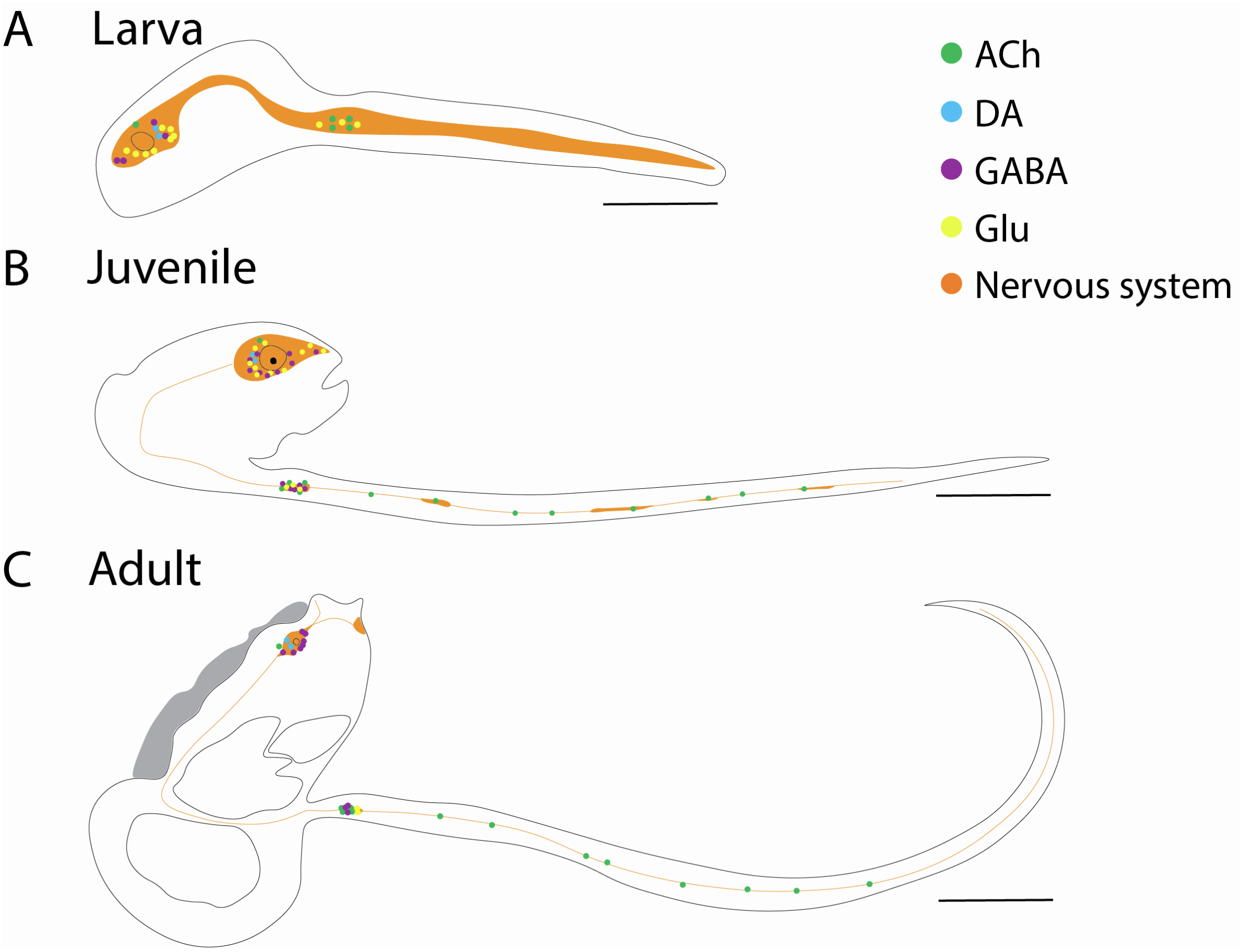

Figure S1: Neurotransmitter expression in Oikopleura across ontogeny (related to Figure 1)

(A-C) Developmental stages analyzed: larva (A), juvenile (B), and adult (C) with neurotransmitter distributions indicated (ACh, acetylcholine; DA, dopamine; GABA, γ-aminobutyric acid; Glu, glutamate). Orange shaded areas indicate the nervous system (modified from Canestro et al.[1]) Scale bars are equal to 20µm (A); 20µm (B) and 500µm (C).

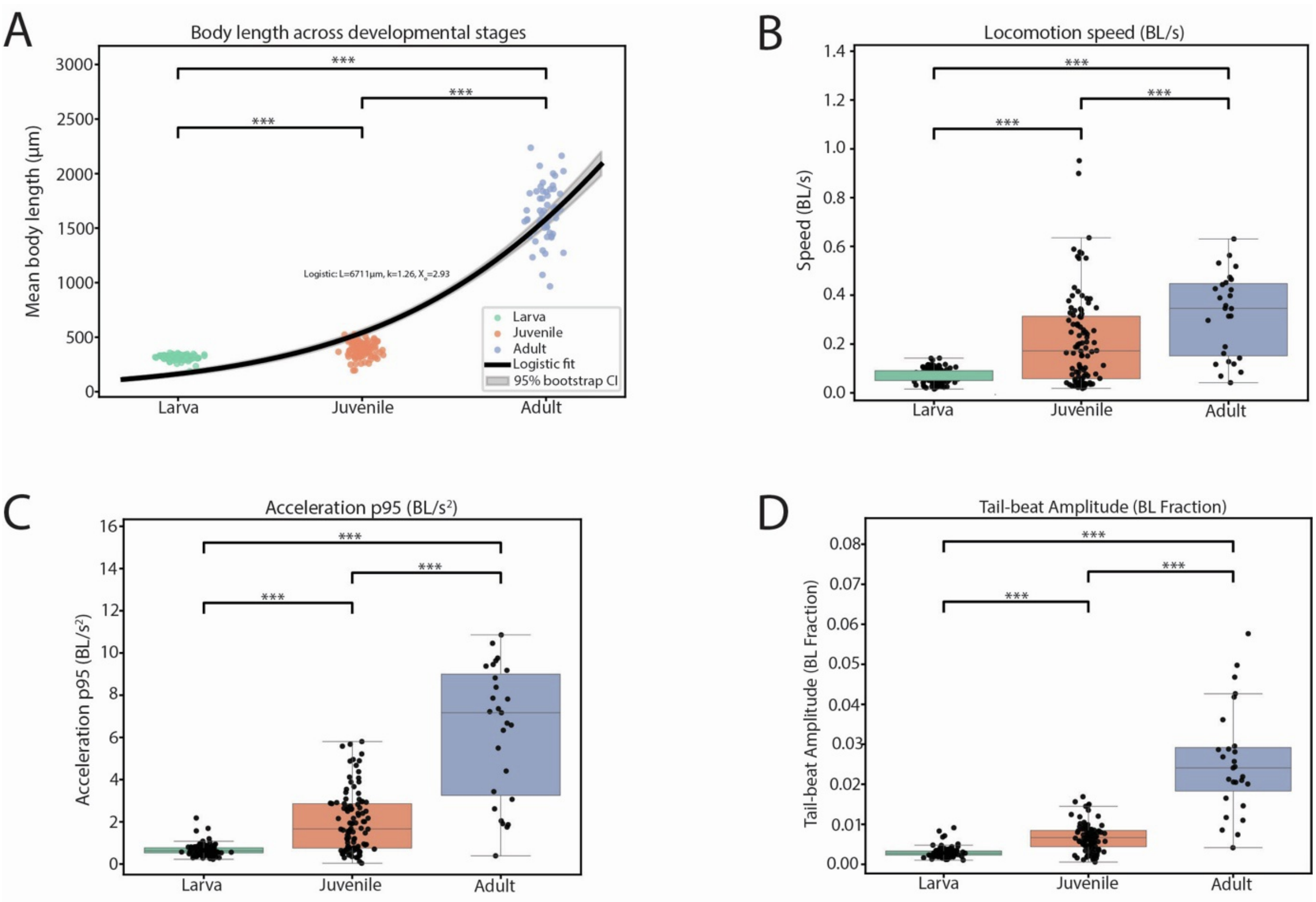

Figure S2: Differences in kinematic features across ontogeny are independent of body length. (related to Figure 1)

(A) Mean body length increases from larval to juvenile and adult stages. Thick black line corresponds to the logisitc fit curve and the grey shaded area indicates +/- SEM. (B) Differences in swimming speed persist across larval, juvenile, and adult animals. (C) Acceleration differs significantly between larvae, juveniles, and adults even after normalisation to body length. (D) Differences in tail-beat amplitude across ontogeny persist despite body length normalisation. For the analysis presented in this figure we used 159 larvae, 100 juveniles and 30 adults (outside the house). Box plots in panels B-D show median (center line), interquartile range (box), 1.5× IQR (whiskers). Statistical analysis: Mann–Whitney test; ***p<0.001. Please see Table S2 for additional statistical analysis details.

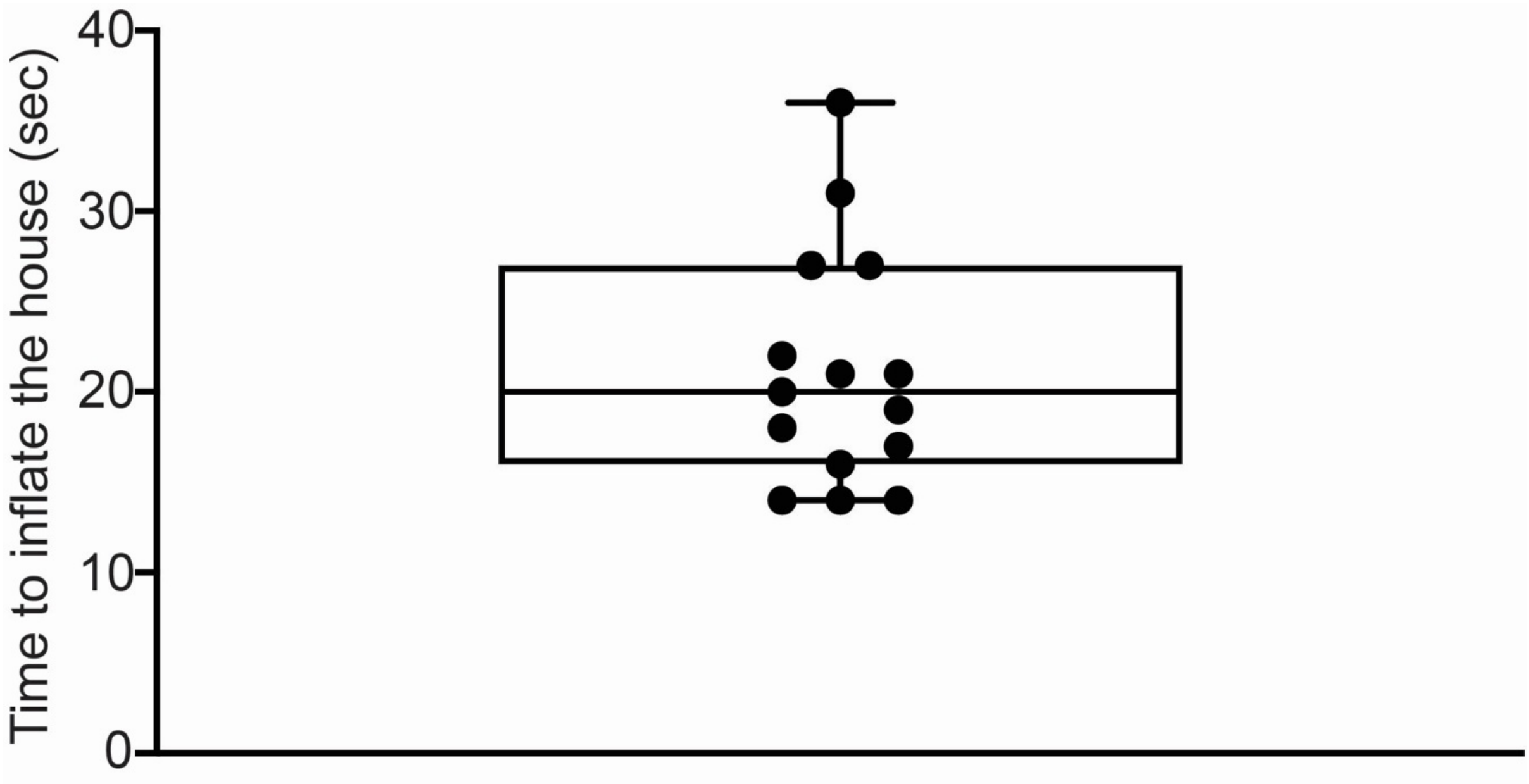

Figure S3: House inflation dynamics in adult *Oikopleura dioica*. (related to Figure 2)

Box plot quantifying the time required for house inflation in adult *Oikopleura dioica* (5 days old). The box plot shows the median, lower and upper quartiles, and minimum–maximum values. Quantification was performed on 15 animals (black circles). Box plot shows median (center line), interquartile range (box), 1.5× IQR (whiskers).

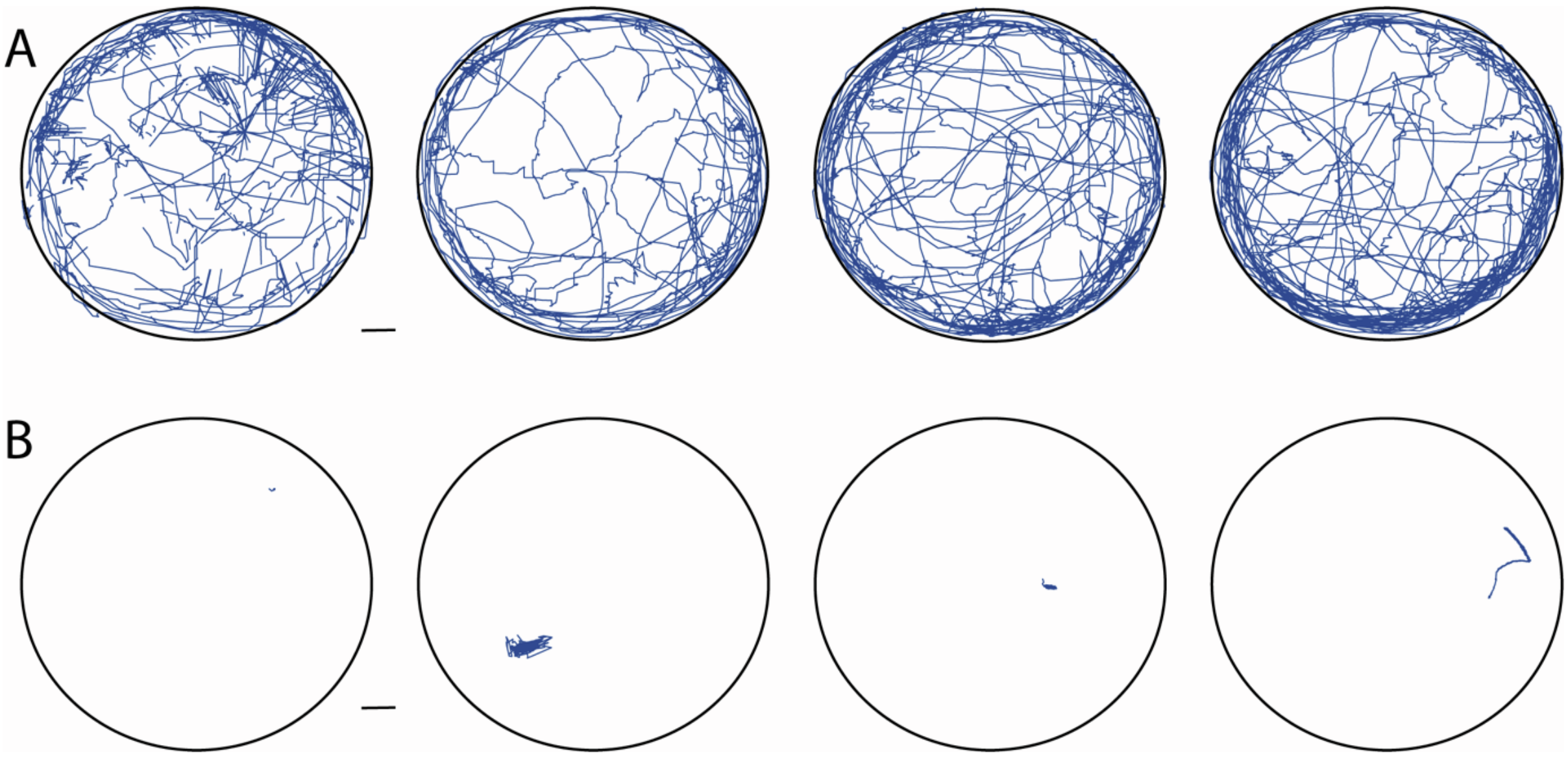

Figure S4: Adult *Oikopleura dioica* outside the house explore a substantially larger fraction of the arena than in-house animals. (related to Figure 2)

Representative 60-s trajectory traces show that adults outside the house (A) exhibit extensive displacement, whereas adults inside the house (B) display minimal displacement. For both panels the scale bars correspond to 1mm.

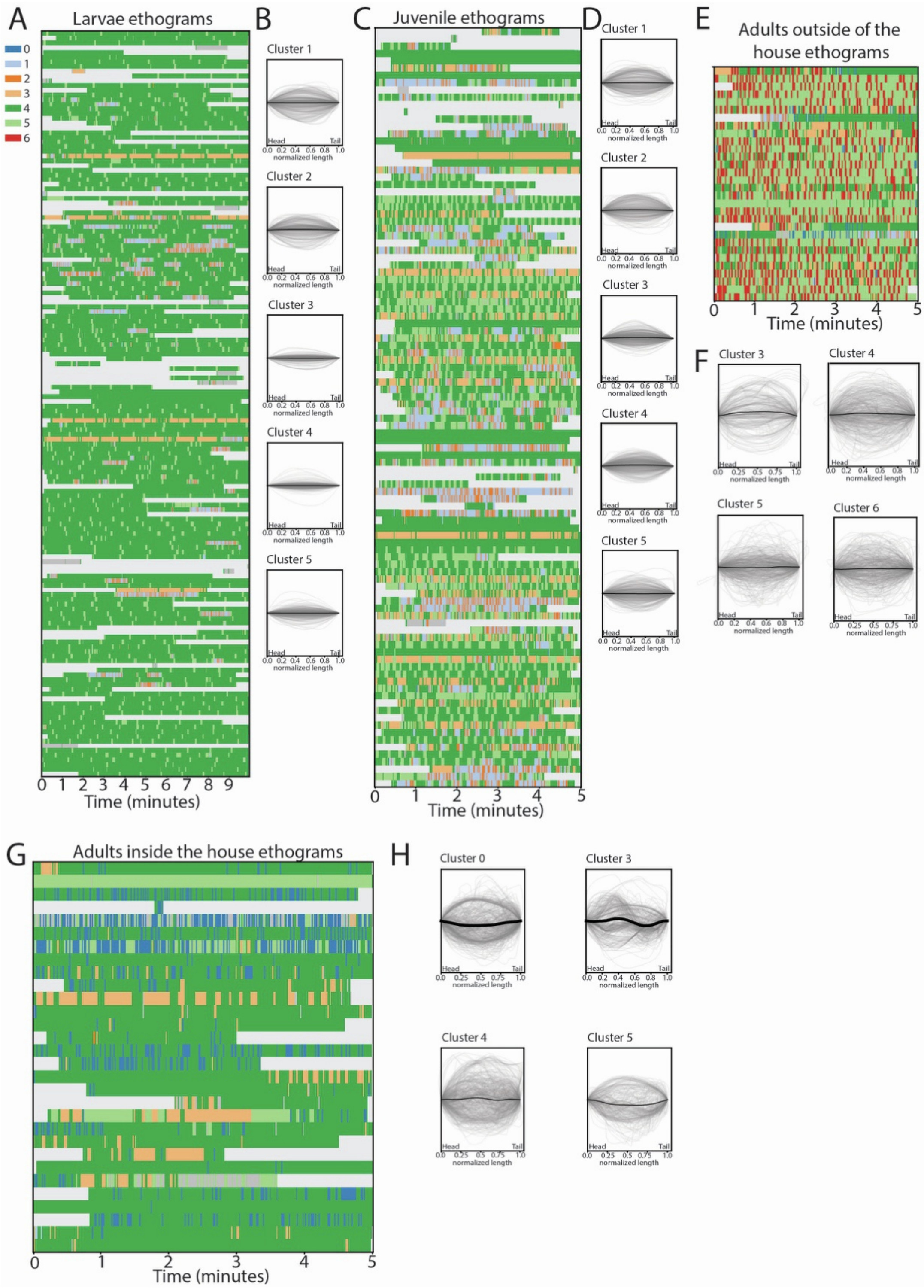

Figure S5: Behavioral cluster assignments over time for individuals across developmental stages and adult contexts. (Related to Figure 4)

(A-B) Larvae. (C-D) Juveniles. (E-F) Adults outside house. (G) Adults inside house. (H) Inside-house. White gaps in ethograms indicate tracking interruptions. Panels B, D F, H show normalized centerline overlays. Black line= mean, grey line= individual animal.

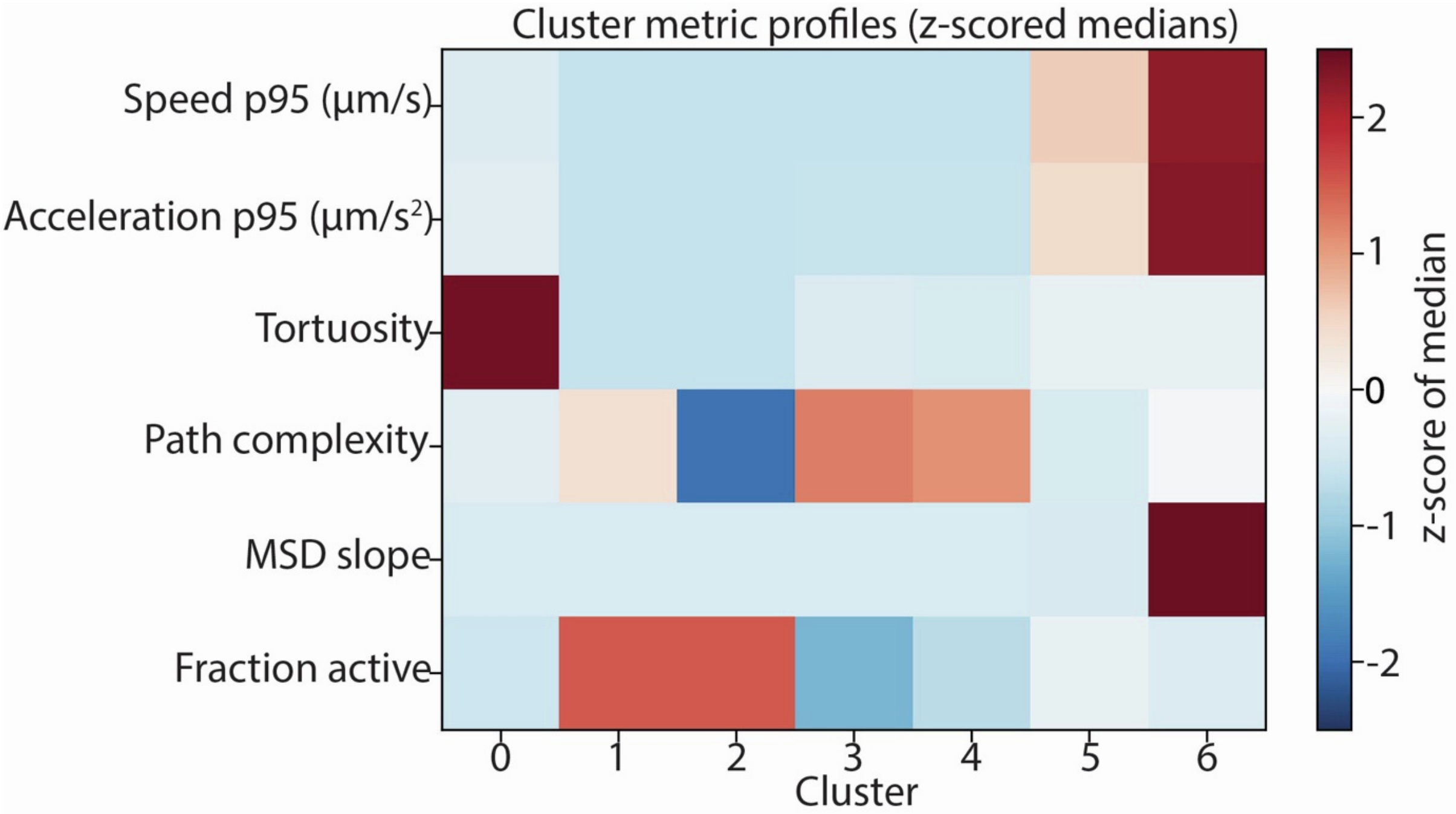

Figure S6: Kinematic and spatial features reveal differences in activity levels across spatiotemporal embedding–derived clusters. (Related to Figure 4) Heatmap showing z-scored median values of kinematic and spatial features—speed, acceleration, tortuosity, path complexity, mean squared displacement (MSD) slope, and fraction active—across spatiotemporal embedding clusters. 57 individuals were used in this analysis. Please see Table S5 for undelrying descriptive statistics.

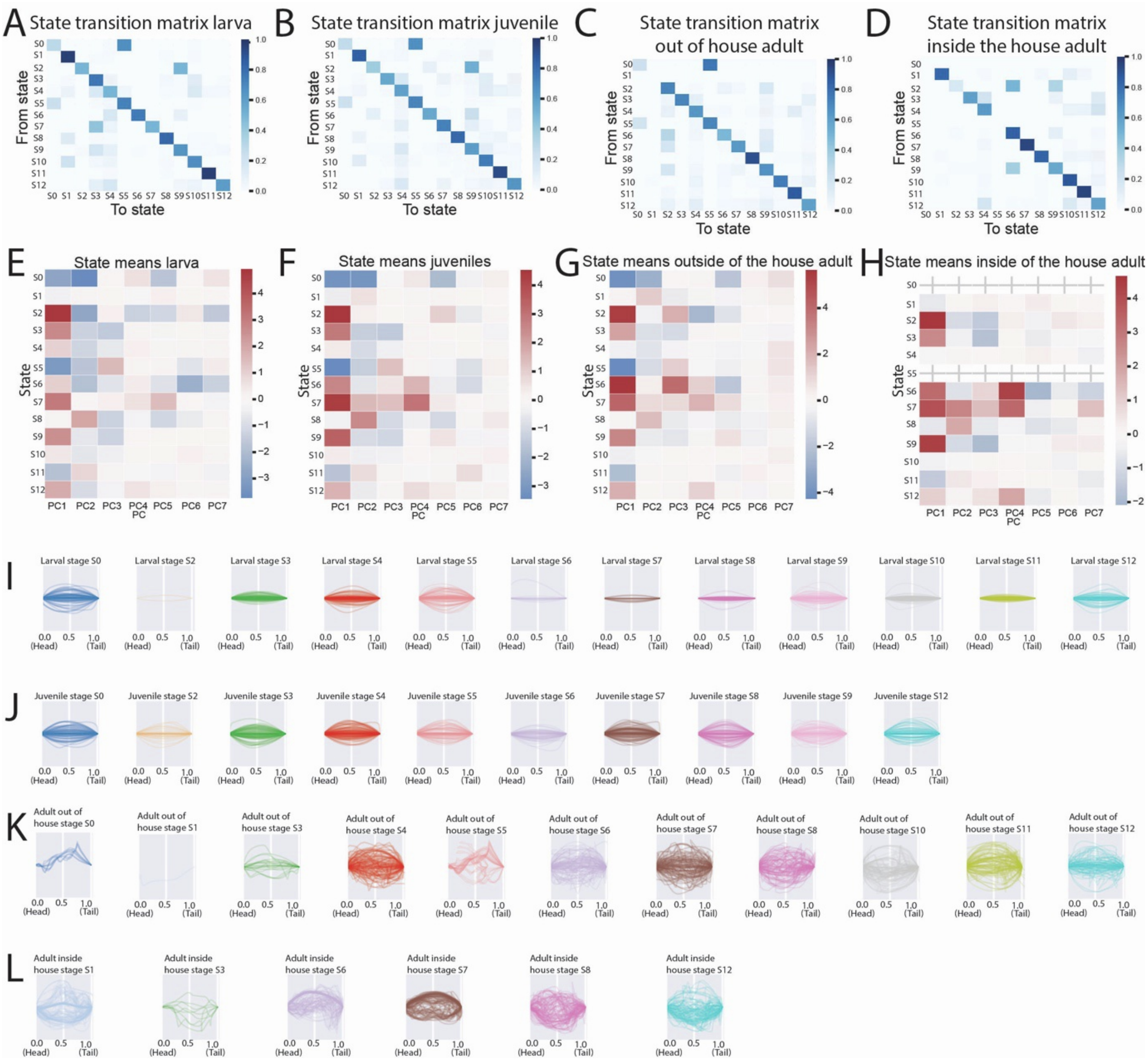

Figure S7: HMM transition networks and state characteristics. (Related to Figure 5)

(A-D) State transition probability matrices for larvae (A), juveniles (B), adults outside (C), and inside house (D). Diagonal = self-persistence; off-diagonal = interstate transitions. (E-H) State emission means in PC space for each condition. (I-L) Representative state-specific centerline shapes showing postural signatures. Developmental progression shows: decreased state persistence (dwell times: 124s to 27s), increased transition complexity, and context-dependent transition networks in adults. For the analysis presented in this figure we used 159 larvae, 100 juveniles and 60 adults (30 inside the house and 30 outside the house).

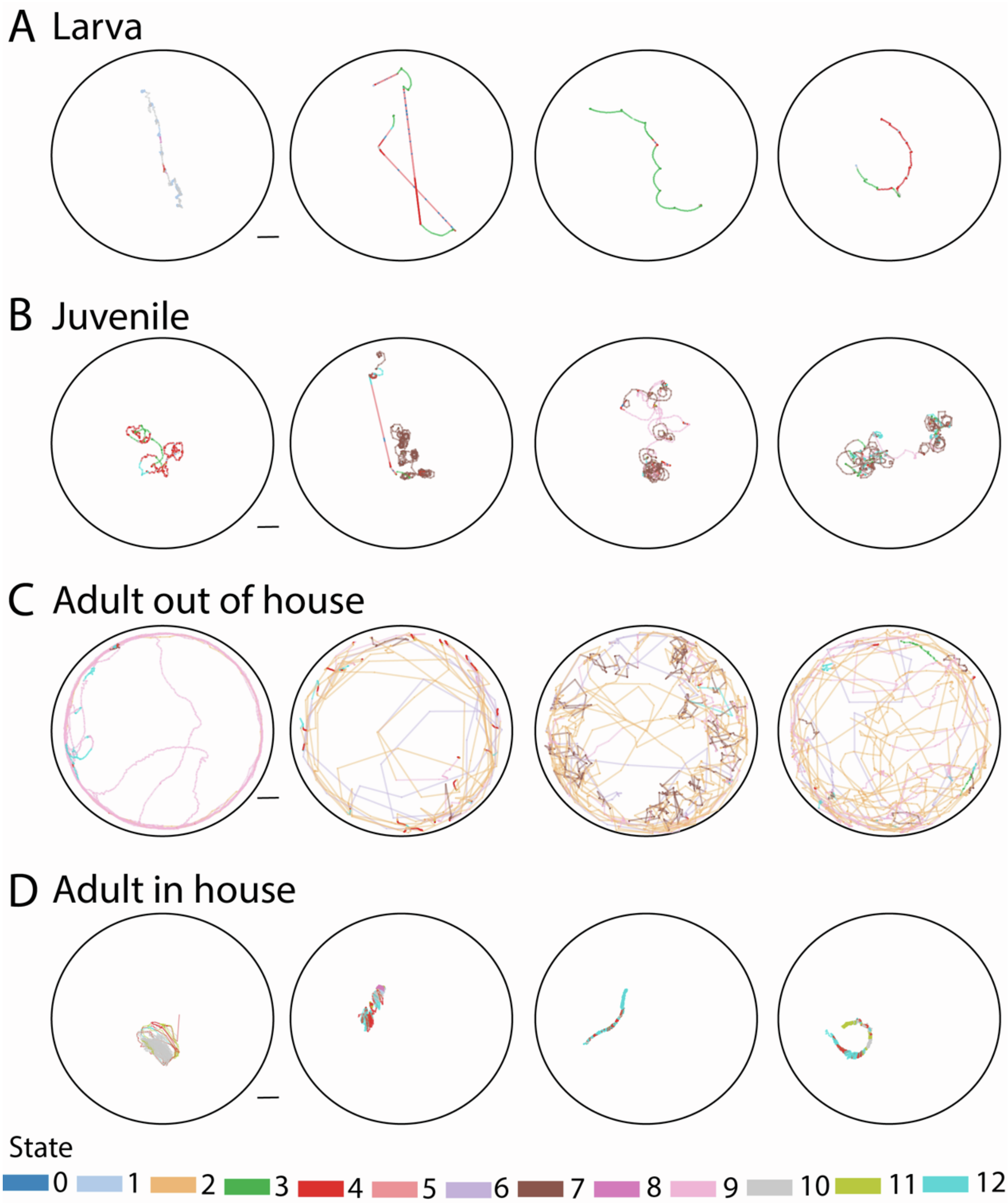

Figure S8: Animal trajectories color-coded by HMM state (Related to Figure 5)
(A-D) HMM state color-coded trajectories inside the behavioral arena for larvae (A), juveniles (B), adults outside (C), and inside house (D). Scale bars in all panels correspond to 1mm.

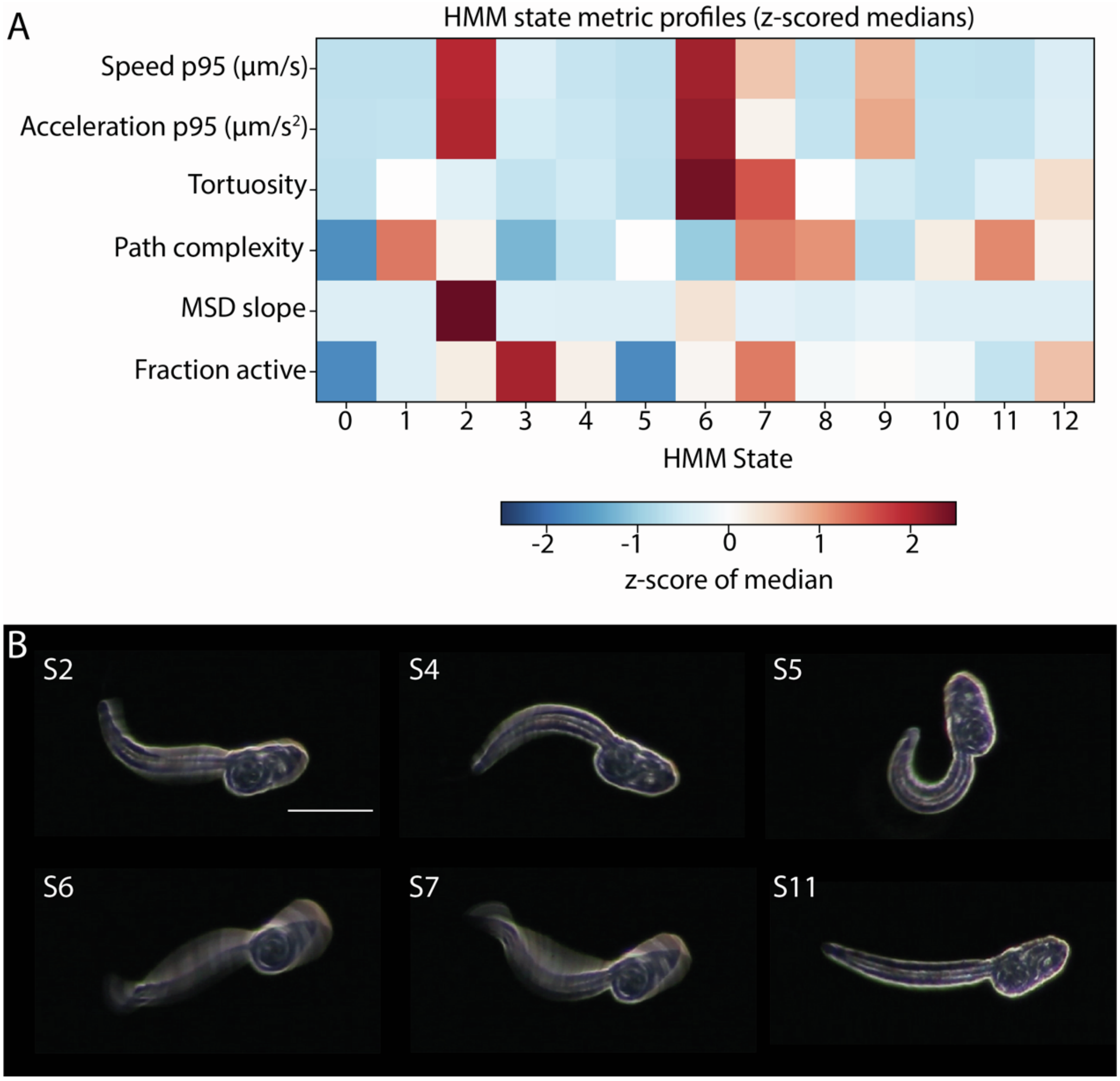

Figure S9: Kinematic and spatial features reveal differences in activity levels across HMM states. (Related to Figure 5)

(A) Heatmap showing z-scored median values of kinematic and spatial features, speed, acceleration, tortuosity, path complexity, mean squared displacement (MSD) slope, and fraction active, across HMM states. For the analysis presented in this figure we used 159 larvae, 100 juveniles and 60 adults (30 inside the house and 30 outside the house). Please see also Table S7 for the underlying descriptive statistics. (B) High-resolution images illustrating example postures of *Oikopleura* larvae corresponding to the identified HMM states. Scale bar in panel B corresponds to 100µm.

REFERENCES

1. Canestro, C., Bassham, S., and Postlethwait, J. (2005). Development of the central nervous system in the larvacean Oikopleura dioica and the evolution of the chordate brain. Dev Biol *285*, 298-315. 10.1016/j.ydbio.2005.06.039.
